# Letters of Recommendation by High School Counselors in Selective College Admissions: Differences by Race and Socioeconomic Status in Letter Length and Topics Discussed

**DOI:** 10.1007/s11162-025-09847-5

**Published:** 2025-07-04

**Authors:** Brian Heseung Kim, Julie J. Park, Pearl Lo, Dominique Baker, Nancy Wong, Stephanie Breen, Huong Truong, Jia Zheng, Kelly Rosinger, OiYan A. Poon

**Affiliations:** 1 Common App, Arlington, USA; 2 University of Maryland, College Park, USA; 3 University of Delaware, Newark, DE USA; 4 University of Massachusetts, Amherst, USA; 5 Pennsylvania State University, University Park, PA USA

**Keywords:** Admissions, Diversity, Equity, Letters of recommendation, Counselors

## Abstract

Letters of recommendation from school counselors are required to apply to most selective universities. We use cutting-edge natural language processing techniques to algorithmically analyze a national dataset of over 600,000 student applications and counselor recommendation letters submitted through the Common Application. We examine how the length and topical content of letters (e.g., sentences about Personal Qualities, Athletics, Intellectual Promise, etc.) relate to race/ethnicity, sex, and proxies for socioeconomic status. We explore whether differences in letter characteristics persist when accounting for additional student, school, and counselor characteristics; letters written by the same counselor; and for students with higher standardized test scores. We found noteworthy naïve differences in letter length and content across nearly all demographic groups, many reflecting known inequities (e.g., longer letters and more sentences on Personal Qualities for private school students). However, the ultimate implications of these patterns hinge on exactly *how* and *when* letters are used in admissions processes (e.g., are letters evaluated at face value across all students, or are they mostly compared to other letters from the same high school or counselor?). Findings reflect the importance of reading letters and applications in the context of structural opportunity, although they do not point to a clear recommendation on whether institutions should keep or discard requirements related to letters or standardized tests. We discuss additional implications and recommendations for admissions policy and practice.

**Supplementary Information:**

The online version of this article contains supplementary material available 10.1007/s11162-025-09847-5.

Numerous inequities exist in college counseling across school contexts and demographic groups (Gast, 2021). Some counselors provide high-quality guidance, but others may discourage students from applying to four-year institutions (Linnehan et al., 2011). Counselors at lower-resourced high schools juggle multiple responsibilities and thus are often limited in the time and energy they can dedicate to college counseling (Woods & Domina, 2014). In contrast, private school counselors dedicate about 65% more time to college counseling than their public school counterparts (Clinedinst, 2019). Despite the recommended student-to-counselor ratio being 250:1, the actual national ratio is much higher (385:1), with major disparities noted between public and private schools (American School Counselor Association (ASCA), 2023).

Despite decades of research on disparities in college counseling (e.g., McDonough, 1997) little is known about the part of the college application process that counselors shape most directly—the counselor letter of recommendation, which is required at most selective institutions of higher education. For years, researchers were limited in their ability to analyze letters at large scale due to the difficulty of gaining access to letters and the human power previously required to code thousands of letters. Earlier work on letters of recommendation within single institutions or individual state networks revealed concerning patterns related to race/ethnicity and socioeconomic status (SES), such as differential topics of discussion, differing strength or positivity of praise, and disparate narratives for student success (e.g., hard work versus innate talent; Akos & Kretchmar, 2016; Rothstein, 2022; Schwarz, 2016). However, due to the aforementioned methodological challenges, prior studies on letters of recommendation have used limited data samples, and results may not generalize to the broader population or different institutional contexts.

With recent advances in comprehensive data collection systems and advanced natural language processing (NLP) methodologies, we have the opportunity to examine how characteristics of counselor letters vary across demographic groups nationally. Research on counselor letters and other non-standardized components of the application is critical to inform ongoing policy conversations about the future of the college application, debates about testing policy, and rising calls for admissions reform. We leverage the most advanced NLP techniques available to analyze a sample of 615,557 counselor letters of recommendation for students who applied through the Common Application (“Common App”) portal during the 2018–2019 and 2019–2020 admissions cycles, asking the following:

1. How do characteristics of letters of recommendation written by counselors, such as length and content, vary by school characteristics (e.g., private/public) and student characteristics (e.g., race/ethnicity, SES)? Do differences in characteristics of counselor letters of recommendation persist when controlling for student, school, and counselor characteristics?
2. Do differences in characteristics of letters of recommendation exist even among those letters written by the same counselor? Do these differences persist when comparing only students with SAT/ACT scores in the 95th percentile or higher?

To preview key findings, private school and continuing-generation students received letters with more sentences, as did students from high schools in wealthier areas. This trend persisted for the latter two groups even when comparing letters written by the same counselor and for students with the highest SAT/ACT scores. Fee waiver recipients received longer letters than non-recipients, with more sentences in their letters about Personal Qualities, likely reflecting contextualization about an applicant’s circumstances. However, these sentences came at the expense of sentences related to Extracurriculars, Academics, and other topics. Private school students had significantly more sentences than public school peers on Personal Qualities, Character Excellence, Intellectual Promise, the Humanities, Extracurriculars, and Athletics. Fee waiver recipients and first-generation students had significantly fewer sentences on Intellectual Promise, Academics, STEM, Extracurriculars, Arts, and Athletics, even when only comparing students from these groups with the highest SAT/ACT scores.

Related to race/ethnicity, Black and Latinx students had letters with fewer sentences overall and fewer sentences on Intellectual Promise relative to White students when comparing letters written by the same counselor, but these disparities did not persist when only comparing students with the highest test scores. However, some disparities related to race persisted even when comparing only students with higher test scores. For example, relative to White students, Black and Latinx students have significantly fewer sentences on Extracurriculars, Arts, and Athletics even when comparing letters written by the same counselor for students with the highest SAT/ACT scores.

## Literature Review

We first address the role of counselors in applying to college and then highlight the role of letters of recommendation in admissions, including how bias and inequity may shape letters. Lastly, we discuss research on letters and patterns related to race/ethnicity. High school counselors play a significant role in supporting the college admissions process (Bryan et al., 2011; McDonough, 1997), helping students obtain SAT/ACT fee waivers, writing letters of recommendation, and providing college advising (Mulhern, 2020). Lower student-to-counselor ratios have been linked with improved test scores as well as increased four-year college enrollment (Carrell & Hoekstra, 2014; Hurwitz & Howell, 2014; Reback, 2010). Effective college counseling is especially impactful for low-income and underrepresented racially minoritized (URM) students, likely because these students have less access to guidance through other means (Mulhern, 2020).

However, not all students have access to effective or supportive counselors. Low-income students of color, especially those at urban schools, report feeling less supported by their counselors in the college application process (Cook et al., 2021; Gast, 2021). Counselors may also subtly or explicitly discourage low-income and Black students, including high achieving Black students, from considering four-year and/or selective institutions (Linnehan et al., 2011; McKillip et al., 2012). Significant disparities in college admissions counseling exist between low versus high SES high schools (Clinedinst & Koranteng, 2017; McDonough, 2005). Private schools typically have a counselor staff that is mostly or exclusively devoted to college admissions counseling, providing highly individualized support and attention with relatively low caseloads of students (Weis et al., 2014). Many affluent students also hire private, non-school affiliated college counselors for additional support (McDonough, 1997). In contrast, some public school counselors have to dedicate more time to issues like discipline, course registration, and social services, lowering time for college counseling (Woods & Domina, 2014).

### Letters of Recommendation in the College Admissions Process

Competitive colleges are more likely to utilize letters of recommendation because they have so many applicants with high levels of academic achievement (Schwarz, 2016), making factors beyond grades and test scores more relevant. In theory, letters can provide deeper insights into who a student is, capturing traits that are not always reflected in GPA or test scores (Kuncel et al., 2014; Oliveri & Ezzo, 2014). Letters can also highlight important contextual information, helping readers better understand a student’s background and context for opportunity (Rothstein., 2022). Letters submitted by counselors provide a unique vantage point since they compare students to a broader range of their peers and/or the student body as a whole, versus teachers, who generally compare students to other students in their classes.

The landmark case *Students for Fair Admissions (SFFA) v. Harvard* (2023), which resulted in the Supreme Court restricting race-conscious admissions nationwide, provided actual data on letters of recommendation for applicants, as well as insight into the role that letters play in ratings used by admissions staff. Letters submitted by counselors received lower ratings than teacher letters among Harvard applicants (Arcidiacono, 2018), possibly because most counselors are comparing students within a larger pool of peers than teachers. At Harvard, letters of recommendation from teachers and counselors were considered in a “personal rating” assigned to applicants, along with materials like the essay (Arcidiacono, 2018; Card, 2017). Higher personal ratings were correlated with a higher likelihood of admission. Studying elite college applicants, Chetty et al. (2023) estimate that about 30% of the admissions advantage accrued by students from the top 1% of household incomes could be attributed to non-academic traits gleaned from evaluation of extracurriculars, letters of recommendation, and other sources, and much of the differential was mediated by private school attendance.

Accordingly, 61% of colleges reported placing considerable or moderate importance on counselor letters when reviewing applications (Clinedinst & Koranteng, 2017). Counselor recommendations were the fourth most important factor in admissions decisions cited, following grades, curriculum strength, and test scores. Some institutions view letters of recommendation as a tool that can help reduce equity gaps in enrollment by providing greater insight into applicants (Oliveri & Ezzo, 2014). However, highly selective institutions that reported weighing subjective factors (i.e., those gleaned from interviews, letters of recommendation, and essays) more heavily had lower rates of Pell Grant enrollment, although there was no evidence of a relationship with URM student enrollment (Rosinger et al., 2021). Private institutions, especially highly selective colleges and universities, place greater weight on such factors (Rosinger et al., 2021).

Disparities in resources between public and private schools can affect actual letters. Analyzing applications submitted through the Common App platform, Nicola and Munoz-Najar Galvez (2022) found that counselors from large public schools were most likely to reuse text in letters of recommendation, likely reflecting limited time and high caseloads. In contrast, counselors from private and affluent schools experience numerous advantages in the letter writing process, as detailed by Schwarz (2016). First, private school teachers and counselors often receive more time and additional compensation (i.e., summer pay) to write letters, helping them to write higher-quality recommendations. Second, private schools have smaller school and class sizes, allowing teachers and counselors to get to know their students better, which can affect letter quality. This dynamic is especially pertinent to counselor letters, given disparities in student-to-counselor ratios (ASCA, 2023). Third, counselors at affluent and/or private schools have more experience writing letters targeted to selective institutions because they have longstanding relationships. They know how to write in a way that will catch reviewers’ eyes, which Schwarz (2016) refers to as “shared language” (p. 184). Counselors and teachers at “feeder schools” (i.e., elite private high schools) can also have established relationships with admissions officers at elite colleges (Schwarz, 2016, p. 34), which often host annual visits and tours for feeder school personnel. As such, admission officers often trust the credibility of the letters written by counselors or teachers that they have established relationships with (Nicklin & Roch, 2009; Posselt, 2018). All of these components make letters of recommendation a vehicle that can perpetuate inequity (Schwarz, 2016), especially if letters are not read within the context of structural opportunity.

### Race and Class in Letters of Recommendation

Counselors themselves may be vulnerable to race and class-related bias, which may have implications for letters. Implicit bias is common within the general population, as well as educational spaces (Chin et al., 2020; Quinn, 2020; Starck et al., 2020). Racial bias is magnified when people have to make split-second decisions (Payne, 2006), and counselors often have limited time to get to know students, especially in larger school settings. Multiple studies document racial inequities in counseling-related issues such as school discipline, academic tracking, and referrals for gifted education (Francis et al., 2019; Grissom & Redding, 2016; Linnehan et al., 2011). In one study, counselors were more likely to recommend community college to high-achieving Black students than White students (Linnehan et al., 2011). Such biases may affect letters themselves, from the language that counselors use to the topics referenced (Kim, 2022).

Patterns related to race and class in letters can be gleaned from research on letters of recommendation for medical residency and other contexts (Brown et al., 2021). Grimm et al. (2020) investigated 2,624 letters written for 736 diagnostic radiology residency applicants in 2015–2016, finding that male and senior rank faculty used more agentic terms such as ethic, confidence, and leadership potential to describe White and Asian/Asian American applicants, compared to Black and Latinx applicants. Examining 2,625 letters for an academic orthopedic residency program, Powers et al. (2020) discovered that letter writers used more standout words (e.g., amazing, exceptional, outstanding, remarkable, superb) to describe White applicants, but described students of color with more grindstone words (e.g., hardworking, dedicated, diligent, organized, persistent). In a study of internship applications, letter writers emphasized White students’ cognitive ability, insight, productivity, and perception while describing non-White students with more communal words that highlighted their positive emotion; trends were consistent regardless of GPA (Houser & Lemmons, 2018).

Several studies document socioeconomic and race-related patterns in letters in the collegiate setting. Chetty et al. (2023) found that students in the top 1%, and especially the top 0.1%, of household incomes were notably more likely to receive the strongest ratings for both counselor and teacher recommendations, even when controlling for standardized test scores. About 36% of students from the top 0.1% of households received a top counselor rating, and 30% for students from the top 1% of households. In *SFFA v. Harvard*, Asian American students received weaker ratings on counselor letters (Arcidiacono et al., 2022), possibly because White applicants to elite institutions are more likely to come from private school backgrounds (44%) than Asian American applicants (24%) (Park & Kim, 2020). In a study of 13,000 letters from teachers and guidance counselors submitted to a selective institution, teacher letters for students from private high schools were longer and generally more positive (Schwarz, 2016). Letters for students of color contained more neutral language, while female students were described more positively. Akos and Kretchmar (2016) analyzed 4,792 letters for applicants to a selective public university in the Southeast. Teacher recommenders were found to use slightly fewer grindstone words (e.g., hardworking, dedicate, diligent, organized, and persistent) when they described Black, Latinx, and Indigenous students. The differing findings from these studies may be partially attributable to the idiosyncratic samples to which researchers could gain access.

More recent research has used NLP techniques (Fesler et al., 2019) to analyze even larger samples. Studying applications from the University of California, Berkeley in 2017, Rothstein (2022) found that letters written for URM students (including low-income, first-generation, and underrepresented racially minoritized students, as well as those from under-resourced high schools) were somewhat distinctive and slightly weaker than those written for non-URM students. Additionally, URM students with average-quality letters received better application outcomes (i.e., higher ratings, higher probability of admission) when their letters were included in the application, but better outcomes were not associated with letter strength. Rothstein (2022) notes that letters may contain information highlighting a student’s context for opportunity, which can help contextualize a student’s achievements. Analyzing letters for 1.6 million students to 800 postsecondary institutions written by 540,000 teachers, Kim (2022) found salient linguistic racial and gender-related trends in letters written by teachers. Overall, Black students’ letters contained fewer positive sentences and slightly more negative sentences. Teachers emphasized Black students’ community engagement and leadership more than their academics, particularly in letters to highly selective institutions. Asian/Asian American students’ letters were slightly more positive than White students and teachers highlighted community engagement, extracurriculars, STEM subjects, and future potential in their letters more so than for White students. Asian/Asian American students' letters contained less emphasis on intellectual promise, but there was no difference in personal/character-based topics (e.g., character excellence, diligence, conscientiousness, commitment) in letters to highly selective institutions.

While the latter studies yield critical insights, no study has sought to analyze large-scale patterns related to race and SES in letters written by high school counselors in a comprehensive national sample and while controlling for additional features of students and their counselors. Given differences between teacher and counselor letters (Arcidiacono, 2018), how counselor letters are vulnerable to manifestations of inequality is largely unknown and speaks to the need for the current study. Previous studies have also been limited in access to student applications, as well as the ability to process millions of applications. Our study combines human qualitative coder insight with NLP techniques that allow us to identify trends within a much larger database, advancing research on non-standardized components of college applications.

## Conceptual Framework and Hypotheses

**Fig. 1:**
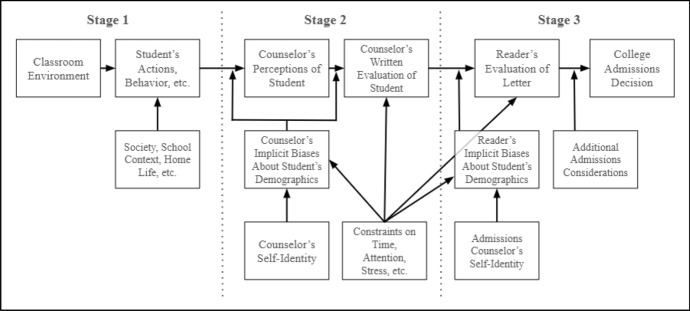
Conceptual framework for counselor letter writing

We adapt Kim (2022) framework delineating the potential role of bias in letters of recommendation from teachers to explain how bias, inequality, and other conditions may relate to letters written by high school counselors, as shown in Fig. 1.

Running along the top-most row from left-to-right, this model conceives of the letter writing process in three stages: the concrete actions taken by and context surrounding the student, the process of writing the letter about the student by the counselor (which is not necessarily a perfect/accurate reflection of the first stage), and the reading of the letter by the admissions professional (which is not necessarily a perfect/accurate reflection of the second stage). These stages ultimately result in some kind of influence on an admissions decision for the student in a specific institutional context. The first stage (concrete actions taken by the student) is shaped by myriad contextual factors, relatively few of which are known to or observed by the counselor, and thus are strongly affected by societal inequities related to race, class, and other factors. In the second stage, what the counselor perceives about the student and writes into the letter is then an imperfect representation of all the contextual knowledge that they are aware of, adjusted by any biases and perceptions that the counselor may hold about the student’s demographic group(s) (Devine et al., 2012; Kang & Banaji, 2006). As shown in the model, such biases and perceptions are further shaped by an individual’s own racial/ethnic identity. For example, Black teachers are known to have higher expectations for Black students than non-black teachers (Gershenson et al., 2016), highlighting how self-identification can influence perceptions of students, which may in turn influence letters. Still, shared identity does not necessarily imply a lack of implicit biases. We theorize that such biases can impact both how a counselor perceives the student’s behaviors and actions, as well as what becomes salient to them about the student while actually writing the letter (e.g., selective memory).

Additionally, we propose that key external conditions influence manifestations of bias and inequity in the letter writing process, as well as characteristics of letters such their length or personalization. These conditions include the time and attention that letter writers can devote to getting to know students and writing letters, as well as external stressors that affect their ability to personalize their work. For example, public school counselors from large high schools are more likely to reuse text in letters (Nicola & Munoz-Najar Galvez, 2022), reflecting how they shoulder large caseloads and have less time to tailor letters. Limitations on time and attention can also exacerbate bias, because having less time to get to know students on a more individual level can result in assumptions being made about a student due to their background, whether positive or negative (Payne, 2006). Finally, many of these same dynamics relating to constraints on time, attention, and implicit biases parallel the reading of the letters by college admissions office staff. As we do not have insight into how the letters we examine in this study are evaluated, we cannot speak to nor account for this stage of the letter process, and include it here only to make evident that limitation, opportunity for future study, and potential practical implications.

In adapting (Kim 2022) model, we control for key variables such as academic performance and college readiness indicators (i.e., reflecting how a student’s actions and behaviors would influence a counselor’s perceptions of a student), as well as a student’s race/ethnicity as an imperfect but relevant proxy for patterns that may reflect bias and inequity. Finally, we consider the role of constraints on counselors’ time and attention by controlling for conditions in high schools that likely influence these dynamics (e.g., public or private, observed student-counselor ratios among college-appliers, etc.). Key limitations include our inability to control for a counselor’s specific racial/ethnic self-identification, as well as complimentary data that could capture implicit bias. However, about 74% of ASCA members identify as White (ASCA, 2023), thus most counselors writing letters are White.

As related to our first research question, we hypothesize that letter length will be longer for private school, White, wealthier, and continuing-generation students, reflecting attributes related to stages 1 and 2 of our model. Stage 1 attributes include the influence of the school environment (especially public versus private); how race and class shape educational resources and classroom experiences; and how race and class shape opportunities to participate in extracurricular activities that counselors may discuss as part of the letter. Stage 2 attributes include how counselors will likely face fewer constraints on time and attention, as well as lower stress levels, in private school settings and in more affluent public high school settings. Additionally, we expect to see greater discussion of topics related to personal qualities, academics, and extracurriculars for these groups, largely due to the factors described in stage 1. Related to our second research question, we hypothesize that these differences will persist even when controlling for letters written by the same counselor and also when comparing only students who have SAT/ACT scores in the 95th percentile or higher of scores.

## Methods

**Fig. 2:**
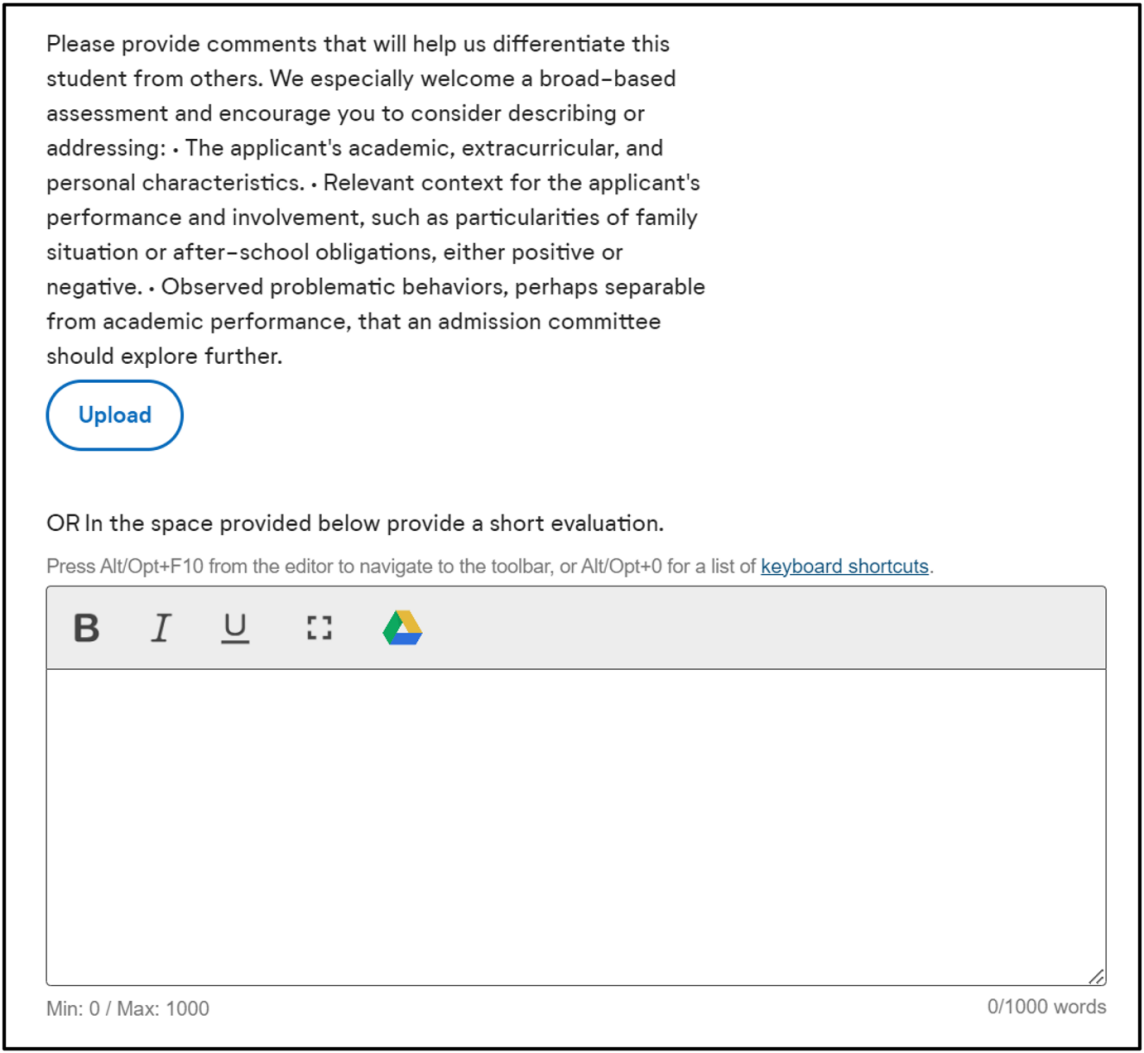
Counselor letter submission interface

Our dataset consists of de-identified applications submitted through Common App during the 2018 (Fall of 2018 through Spring of 2019) and 2019 (Fall of 2019 through Spring of 2020) application cycles.^1^ These application data include nearly all submitted components for each student, such as academics, course-taking, standardized test scores, and demographic information. Moreover, the dataset includes all information submitted via the Common App on the student’s behalf, including their counselor recommendation form and letter. The counselor recommendation form asks a series of questions about a student’s academic background (e.g., class rank and GPA, largely serving as reinforcement and verification of the academic data the student submits themselves), whereas the letter is a more open-ended space for counselors to submit their evaluations of the student.^2^ Figure 2 displays the interface that counselors navigate for the letter submission process.

Counselors can submit their letter through two distinct means: a document upload (which is immediately converted to PDF format) or an open-text response field. Counselors can decline to submit a letter on the student’s behalf even if they complete the rest of the recommendation form. For this study, we only analyzed letters uploaded as PDFs, which constitute about 90% of the counselor letters submitted via Common App, to avoid comparing across these two meaningfully distinct formats. We find some potentially meaningful differences between PDF letters and letters uploaded via open-response (e.g., students with open-text letters are more likely to attend public school, be low-income, and be first-generation) and further discuss the implications of these issues in the online appendix. In short, it may be that our estimates of disparities are perhaps slight *underestimates* if the open-text letters are coming from public school students with lower resources and tend to be shorter (due to an enforced word limit). Regardless, because these open-text letters are proportionally much fewer in number, the letters would need to be *radically* different to meaningfully change the estimates we present here in gesture.

### Overall Study Sample

We focus on students in these years who submitted a complete application (hereon referred to as “applicants”) to at least one selective four-year institution (admit rate of 40% or lower) in the 2018–2019 and 2019–2020 cycles. Admit rates came from the 2019 Integrated Postsecondary Education Data System. We limit our study to domestic applicants^3^ with a complete counselor recommendation letter (in PDF format) of substantive length^4^ submitted to focus on trends in the U.S. context. These restrictions can cumulatively be thought of as attempting to focus our study (and thus the external validity of our ensuing results/findings) on characterizing reasonably complete counselor letters written for domestic students who successfully applied to a selective institution. Our overall sample thus contains 624,108 total applicants (and corresponding counselor letters), or approximately 35% of domestic applicants on the Common App platform during the 2018 and 2019 application cycles (or 29% of all applicants, domestic and international combined).^5^

### Data Splitting

As a final stage in preparing our study sample to improve the internal validity of our results, we performed a procedure known as “data splitting.” Research on text data is especially vulnerable to threats like p-hacking and similarly motivated analyst decision-making because cleaning and modeling text data is necessarily a bespoke and contextually-driven process. For example, text data may include certain undesirable data artifacts like school mottos embedded in a PDF’s header; removing these data artifacts requires systematic cleaning code that is necessarily trial-and-error and idiosyncratic (even “hacky”), and can be difficult to evaluate for its true effect on the data or analyses downstream. Even in best-case scenarios, analysts can unknowingly “bake in” (or, conversely, “bake out”) a desired or expected data relationship due to text cleaning and modeling decisions.

Per recommendations from Egami et al. (2022), we attempt to counteract these concerns by using a “development” and “analysis” split with our data sample (sometimes instead referred to as “training” and “testing” in data science and machine learning). We randomly split the text data into two groups; on expectation, text formatting issues and other data artifacts that need to be addressed should then be evenly distributed across these two groups. Rather than iteratively create our text cleaning and modeling code on the entire dataset altogether, we *develop* these processes only using the *development* subsample. Only when the entire cleaning and analytic pipeline is completely finalized (to include the procedures for hypothesis testing and regression analyses we intend to run) do we then feed the *analysis* subsample through this same pipeline *without any alterations*.^6^ This approach prevents several of the aforementioned issues, as text cleaning and modeling needs to be sufficiently generalizable enough to apply to text data never seen before (as long as it is presumably similar in form and style to the development text data given the randomization process).

The exact methodology of the split is highly consequential. As we ultimately deploy a counselor fixed effects regression analysis strategy, it is crucial to maximize cell sizes at the counselor level. If we randomize at the unit of *letters* into development and analysis subsamples, this approach would split up a given counselor’s letters into each subsample and reduce our power to detect relationships when using counselor fixed effects in the final analysis subsample. Instead, we randomize at the unit of *counselors* into the development and analysis subsamples, so if a counselor is randomized into one subsample, so are all of their letters and students.^7^ We stratify this randomization procedure by the number of PDF letters and open-textresponse letters they wrote in our sample to ensure that lower- and higher-volume counselors for both letter types were represented in both subsamples.

Finally, we randomized 10% of counselors into the development subsample and 90% of counselors into the analysis subsample. This is ultimately an arbitrary decision, but the goal is to maximize the size of the final analysis subsample while still maintaining sufficient variation and volume in the development sample to adequately capture the breadth of text data idiosyncrasies and issues. Given the overall size of our dataset, 10% should be more than adequate, representing 59,776 letters from 4,707 counselors across 3,859 high schools. This same split ratio also seemed to work well in parallel related work on teacher recommendation letters (author omitted).

#### Final Analytic Sample

As a result of the data splitting process, 59,776 of the 624,108 total applicants were randomized into the development subsample, while the remaining 564,332 were randomized into the analysis subsample. All results and analyses discussed and displayed in the main narrative were conducted using the analysis subsample unless otherwise noted; parallel tables and figures for the development subsample are available upon request. Table 1 displays summary statistics for the 2018, 2019, and pooled (i.e., both years) sample.

**Table 1:**
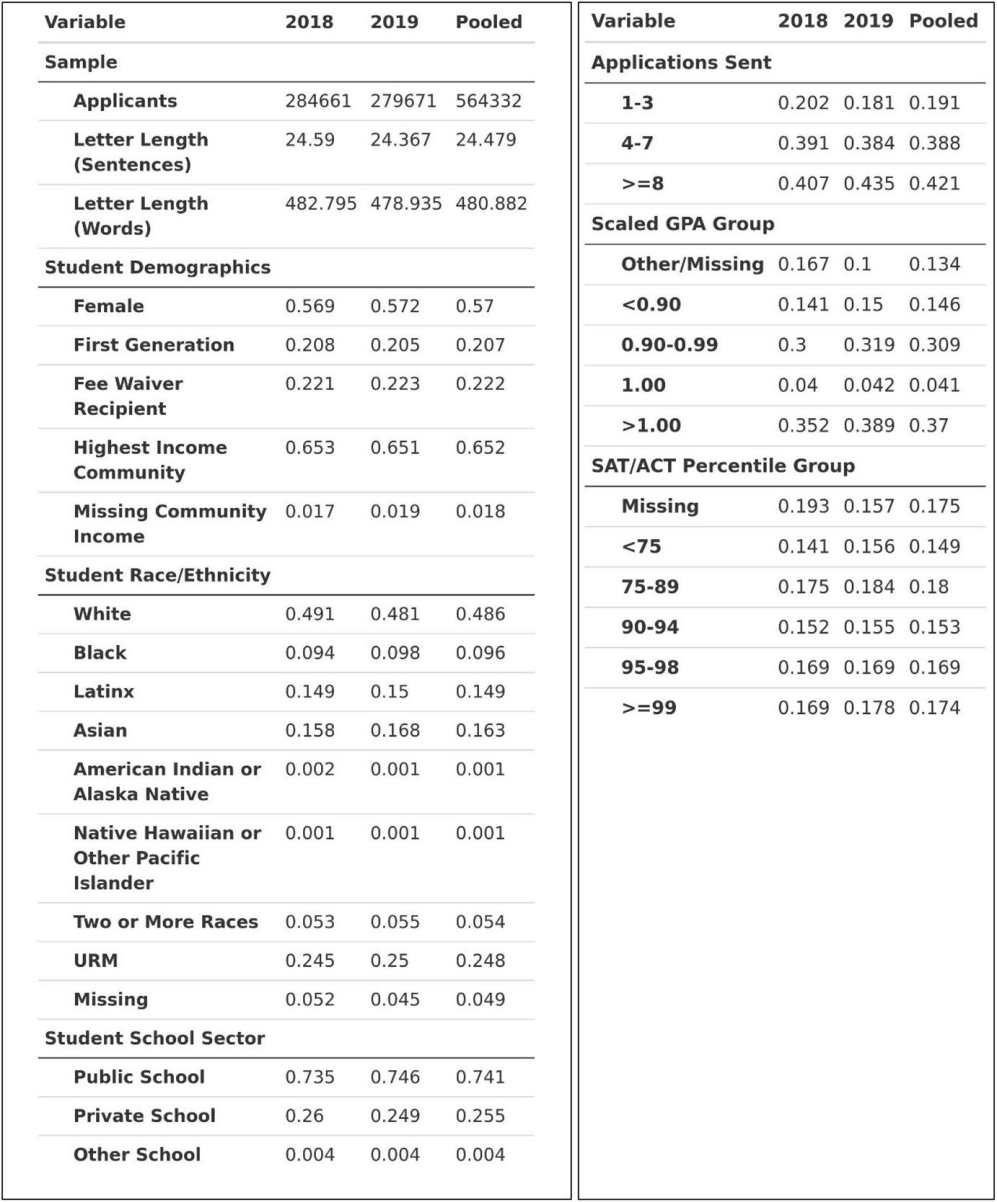
Applicant sample descriptive statistics

The analytic sample is skewed slightly female at 57%, and only 21% of the sample identified as first-generation.^8^ To examine income levels, we rely on two separate measures. First, we use the Common App’s primary measure of low-income status, eligibility for a Common App application fee waiver,^9^ which about 22% of the sample received. Because we are also interested in *high* income status, we merged in ZIP code level median household income data from the U.S. Census to create a rough proxy for each applicant’s community income level. To simplify the measure, we created a binary measure for whether an applicant lives in a ZIP code in the top quintile of ZIP codes with respect to median household income; importantly, this indicates *community* income level, rather than *individual* income level. We calculate top quintile nationally, rather than state-by-state, to better align with existing measures used by Common App. This approach reduces the share of students considered high-income in lower-income states, but better reflects the dynamics of the largely national application markets of the selective institutions of our sample. The majority of applicants (65%) in our sample come from high income communities. About half of our sample identified as White; about 25% of the sample identified as being from a URM group.^10^ About 74% of applicants went to a public school, while 26% went to a private/independent school.

Each applicant’s counselor letter was an average of about 24 substantive sentences, representing a total of 13,814,613 substantive sentences in our dataset. This sample of applicants also tended to submit greater numbers of applications: 19% submitted only 1–3 applications; 42% submitted 8 or more. (Students can submit up to 20 applications per season). Applicants submitted cumulative GPA with their GPA scale; we created a common “scaled GPA” where a value of 1.0 indicates the top of their grade scale (e.g., a 4.0 on a 4.0 scale).^11^ The vast majority of our sample applied before the start of the COVID-19 pandemic; thus, a full 82% of our sample submitted an SAT or ACT score. Over 17% reported a score at the 99th percentile or higher, and about half reported a score at least at the 90th percentile. Table 2 shows descriptive statistics related to the *counselors* in our sample, split by year and pooled.

**Table 2:**
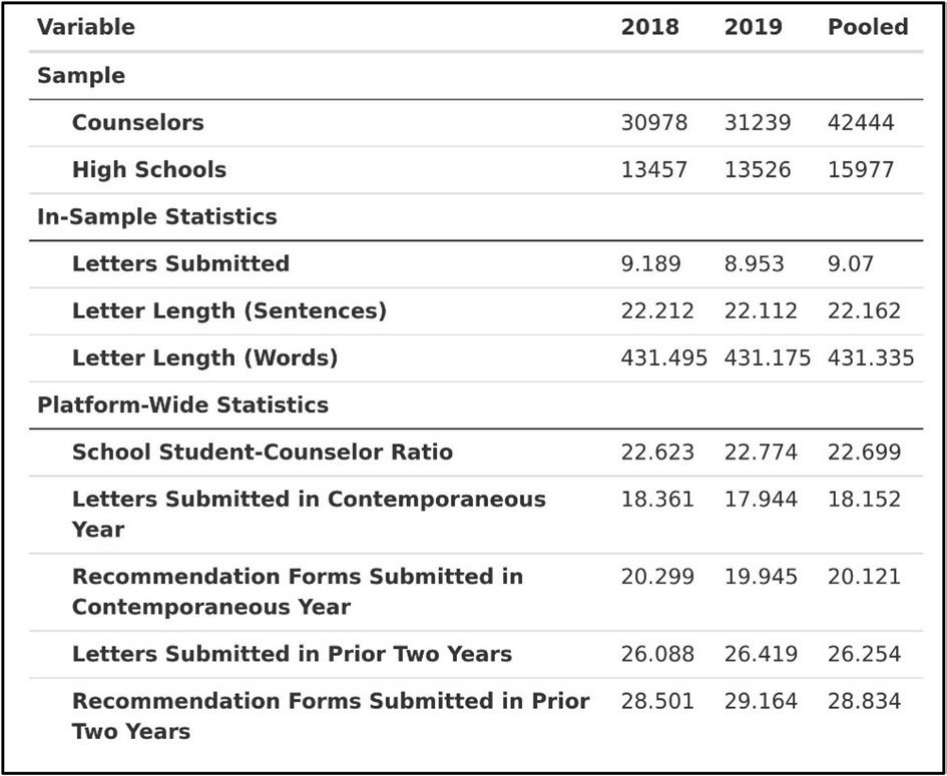
Counselor sample descriptive statistics

The sample has 42,444 distinct counselors from 15,977 distinct schools.^12^ Counselors in our study sample wrote an average of 9.07 letters of about 22 substantive sentences in length.^13^ Zooming out to the full Common App dataset, the average counselor in our sample was embedded in a high school where about 23 students who submitted a completed application for every counselor on the platform. This number differs from nationally reported student-counselor ratios because it only reflects data from Common App for (a) students from a school who are applying to college via the Common App, and (b) counselors at a school who are completing recommendations on the Common App.^14^ Moreover, the average counselor in our sample wrote a total of 18 letters in a given year when looking across all applicants, out of a total of 20 recommender forms submitted. These statistics give a sense of overall counselor “burden” or “load” in a given year (i.e., how their time may be split across many students). We can also calculate how many letters and forms a counselor completed on the platform over the *prior two years* to get a sense of counselor “experience” instead.^15^ On average, a counselor in our sample wrote 26 letters across all students on the platform out of 29 completed recommendation forms.

### Text Analysis Approach

We deployed a two-stage approach for analysis. First, we used an NLP technique known as “topic modeling” to assess the extent to which each letter discusses various substantive topics of conversation. Second, we analyzed each topic as the outcome of a regression while controlling for a variety of student and counselor characteristics.^16^ To explain topic modeling, consider a scenario where we had an infinite number of researchers to read and analyze every letter in the sample. The team would first review a random subset of letters to identify codes and themes in the data. Then they could develop a tentative codebook and framework for identifying when a certain code or theme is surfacing in the data. Next, the team could review a new sample of letters to apply and practice using the framework, updating it to reflect new insights. Once the framework has been *solidified* and *harmonized*, readers could code and analyze the remainder of the letters. Theoretically, counts for each occurring code could be analyzed in a more quantitative manner via regression analyses or other techniques.

While we lack infinite researcher capacity, modern NLP techniques are beginning to offer approximations of this process using topic modeling in conjunction with a Computational Grounded Theory framework (Nelson, 2020). In this context, we deploy a specific implementation of topic modeling from Grootendorst (2022) dubbed “BERTopic.” This particular implementation leverages the most recent advances in NLP (the “transformer” neural network architecture per Vaswani et al., 2017) that allow for more contextual analysis of the meaning of a given word, phrase, and sentence, which better (but not perfectly) captures important language nuances like negation, sarcasm, and multiple word definitions beyond word frequency based approaches such as structural topic modeling (Roberts et al., 2019) or Linguistic Inquiry and Word Count (LIWC; Tausczik & Pennebaker, 2010). On an intuitive level, BERTopic attempts to first “read” each sentence of text provided to it, translating the sentence’s meaning into numbers by characterizing it across hundreds of numeric indices from 0 to 1.^17^ Once this process is complete, the algorithm clusters sentences with similar numeric indices together in this multi-dimensional space through standard clustering procedures (in this case, HDBSCAN as developed by Campello et al., 2013). A strong assumption of the algorithm is that sentences clustered together because of these numeric indices will also share some interpretable or substantive commonality in topic of discussion (e.g., “athletics” versus “community service”). Once the sentences are clustered, we as human analysts must attempt to assess the extent to which this assumption seems to hold true in the output of the algorithm: do sentences assigned to a given cluster actually “hold together” in any interpretable way? Then, pending these checks, what is the substantive topic of discussion for a sentence assigned to a given cluster? If not, the BERTopic algorithm can be adjusted in a variety of ways, as there is no single “best” set of parameters to deploy for a given set of text data. In a process that loosely mirrors the solidification and harmonization steps in the “infinite researchers” hypothetical, a human analyst must iteratively and manually “fine-tune” the BERTopic parameters over a series of several attempts to maximize the extent to which the topical framework that BERTopic has created seems to align with substantively interesting and relevant themes to *humans* in the data.

Ultimately, we can use BERTopic’s output to first identify major topics across counselor recommendation letter sentences, and then identify which sentences fall into which topics.^18^ This parallels the sort of output produced by the aforementioned hypothetical scenario with infinite researchers, facilitating statistical analyses like regression and comparisons across populations. BERTopic should never be thought of a “drop-in” replacement for rigorous qualitative reading, and this sort of approach will never be able to match the nuance, care, and contextual understanding of a human on a case-by-case basis. Even so, we lean on BERTopic to balance the need for scale and nuance within the realm of feasibility. In applying this approach to our data, our research team constructed a single unified coding scheme for topics surfaced by BERTopic – informed by over a dozen model iterations and extensive manual reading of sentences to exhaustively account for all common topics we repeatedly saw across readings and model runs. With this codebook, we ultimately evaluated each BERTopic model on two key dimensions: the extent to which it was able to adequately capture topics present in the data (i.e., to what extent did it surface the same topics we identified in our codebook), and the extent to which its judgments aligned with a trained team of human coders looking at the same sentences (i.e., how often does the algorithm agree with what a human would say about a given sentence’s topic?). We focus on sentences as the main unit of topical classification (rather than words, paragraphs, or whole letters) because it is the most discrete unit where we can reasonably detect a salient topic of conversation. Counselor letters in this context tend to change topics rather quickly, so whole-letter topical analysis would likely be inappropriate. Paragraphs would be ideal, but counselor letters (unlike teacher recommendation letters) tend to have more complicated and difficult-to-parse formatting (bullets, long paragraphs, lists, tables, etc.) and, on review of random subsamples of full letters, tend not to follow traditional paragraph flow (e.g., topic sentences, single topics of discussion per paragraph) even when using clear paragraph structures.

To address to what extent did the algorithm surface the same topics we identified in our codebook, Table 3 displays the codebook scheme we created for topics in our dataset, as well as selected keywords that our final BERTopic model found to be highly representative of each for illustrative purposes. Each shaded partition corresponds respectively with one of the broad categories of topics in order: Academics, Extracurriculars, Personal Qualities, and Other.

**Table 3:**
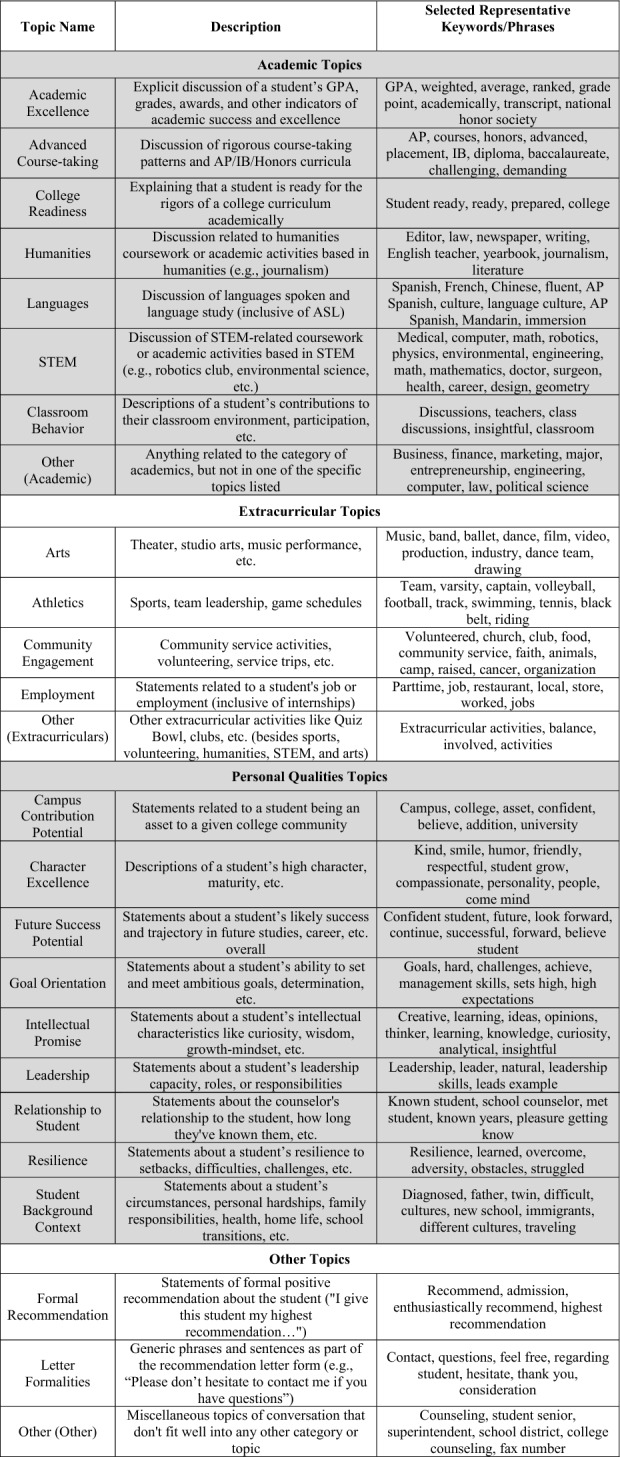
Topics of interest in counselor letter data

There was no topic in our codebook not represented in the final BERTopic model.^19^ To the question of “how often does the algorithm agree with what a human would say about a given sentence’s topic?,” we conducted a rigorous human-algorithm validation process to better understand how the algorithm’s output compares to that of a human reader. We trained six researchers in the use of our codebook to classify real letter sentences from the development subsample data. We then created a stratified random subsample (stratified on student sex, URM status, and public/private school attendance) of sentences from the development subsample that all researchers examined and classified in their own judgment. There were 100 “common” sentences that all six researchers coded, and an additional 400 that were coded only by one researcher each.

Detailed information for inter-rater reliability (IRR) from the human team vis-à-vis the algorithm’s performance using Light’s Kappa (Hallgren, 2012) can be found in the online appendix. Overall, results showed that the algorithm performs well in the context of natural, expected disagreement among humans about how sentences should be categorized. These validity examinations should not conceal the fact that all results in this paper hinge quite firmly on the nuances and idiosyncrasies of this specific model, and further study is required to understand how best to make results more robust to modeling decisions in the NLP pipeline. One caveat is that results with respect to race/ethnicity should be approached with caution given some of the IRR differences discussed in the appendix. In short, our IRR both with *and* without the algorithm was meaningfully higher for URM students versus non-URM students. This could be an artifact of the 100 sentences we ultimately sampled, in that there just happened to be more ambiguity in the sentences from non-URM students by chance, resulting in “true” grounds for disagreement, or due to systematically different styles of writing when counselors write about non-URM students that produces greater ambiguity or complexity in interpretation. The trend seems unlikely to be driven by biases present in our human readers or the algorithm, as readers had no access to student demographic information throughout this process (besides student pronouns used in the sentences). We also manually verified that there were no obvious clues about student race/ethnicity in the sentences themselves. These diagnostics ultimately point to idiosyncrasies in the randomly selected data rather than systematic biases in our rating systems, but we welcome additional interpretation, hypotheses, and future research on this front.

### Regression Analysis Approach

Once each sentence of each letter has been analyzed by BERTopic, we then have an estimate of how many sentences in each student’s letter discuss each topic in Table 3. We treat these values as the outcomes of regression models to assess whether some topics appeared in letters more often for students in one demographic group versus others. We display results from five models. In Model 1, we only control for a single demographic characteristic (e.g., race) and some non-demographic student traits: whether the student was a senior, whether they attended multiple high schools, and whether their letter had a substantial proportion of its text removed during the text cleaning process. This allows us to examine whether there are demographic differences in letters at a broad population level, but does not attempt to control for any other variables. Model 2 is identical to our first, except that we control for all student demographic characteristics (e.g., race/ethnicity, SES) in the same model.

Model 3 controls for demographic characteristics, school and counselor characteristics (including all the Platform-Wide variables in Table 2, e.g., student-counselor ratio), and private school attendance. This model reflects the question: Do we observe demographic differences in the content of letters when accounting for things like a counselor’s past experience in writing letters, current load of writing letters in a given year, and private school attendance? This model helps us explore whether observed differences related to race, sex, and SES in letters could be driven by access to school resources and counselor staffing. In Model 4, we account for all of the variables in Model 3 except for private school attendance, and also deploy more restrictive counselor fixed effects. As schools are fixed within counselors, we can no longer account for school characteristics like sector—hence, the omission of private school attendance in Model 4. Overall, we aim to explore whether we observe demographic differences in letter content even when focusing on letters written by the *same counselor* among high-scoring students. This approach helps better isolate *unobserved* characteristics about the school and counselor such as counselor race/ethnicity and sex, a counselor’s tenure in a given school, and so on. Of note, the counselor fixed effects approach changes the *effective* sample size for the estimation of demographic differences, and changes how we should interpret the *external* validity of the results: A difference in letters between URM and non-URM students can only be estimated among those counselors who wrote letters for both URM *and* non-URM students. Thus, this approach drops counselors who only wrote letters for one group *or* the other; as such this estimate cannot be thought to include the absolute extremes in terms of student demographic composition for counselor caseloads.

Finally in Model 5, we control for the same variables as in Model 4, but focus solely on the on the subset of students who reported a test score in the 95th percentile or higher (roughly a 1430 or higher out of 1600 on the SAT for the 2018–2019 administrations), which we interpret as a subsample of students who are more competitive at selective institutions. The intention here is to non-parametrically and parsimoniously attempt to control for a variety of observed and unobserved student academic characteristics like GPA, advanced course-taking, and so on – without making strict and likely untenable assumptions about the functional form relationships between these academic characteristics and letter characteristics. Put another way, in this model, we are asking: *Do differences in letter content still exist when focusing on letters by the same counselor, for students with high SAT/ACT scores?*

For our analysis, we use these regression models to examine dependent variables related to letter length and content. First, we examine the outcome of overall number of sentences (letter length). We were curious to examine length given questions about whether students from certain backgrounds (e.g., White, more affluent, private school context) would receive longer letters or not. Second, we examine the number of sentences related to key broad topic categories (Personal Qualities, Academics, Extracurriculars) as dependent variables to see if differences in letter length are driven by one broad category more than others, as well as to understand predictors of each letter content outcome. Each broad category reflects key areas prioritized in selective college admissions, in that many selective institutions organize ratings around these topics. For example, *SFFA v. Harvard* revealed that admissions officers assigned personal, academic, extracurricular, and athletic ratings to each applicant (Card, 2017). (In our analysis, athletics is included as a subtopic under the broad category of Extracurriculars.)

Table 4 displays this hierarchy of decomposition, as well as the specific topics identified within each of the broad categories. For example, within the Personal Qualities broad category, counselors wrote sentences related to subtopics like Character Excellence, Intellectual Promise, and Leadership. Importantly, a lack of disparity in one aggregated “layer” of this analysis does not necessarily preclude the possibility of disparities in the decomposed “layer.” For example, it could be that some students receive letters with many more sentences about Academics and fewer sentences about Extracurriculars, while other students receive letters with many more sentences about Extracurriculars and fewer sentences about Academics. The overall sentence length would appear similar between the two groups, masking this important difference.

**Table 4:**
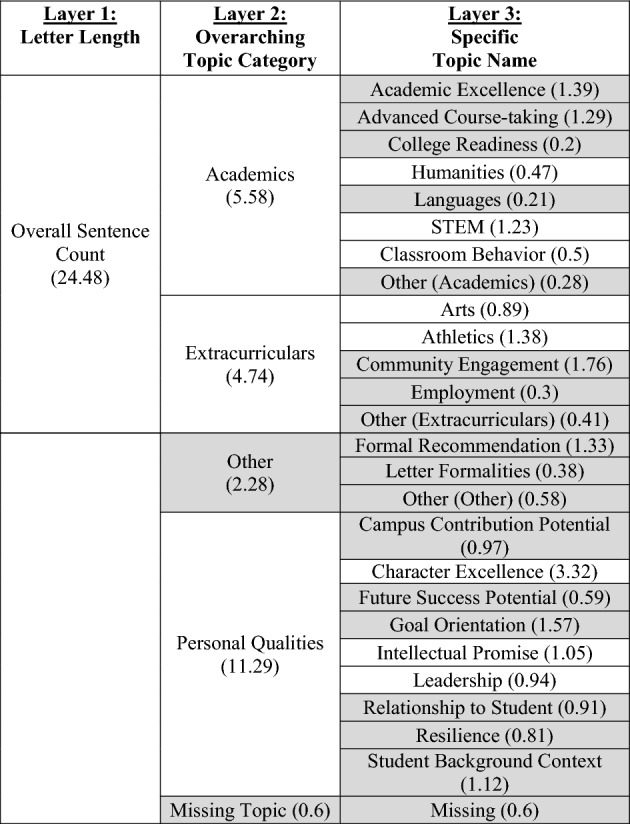
Letter content measure decomposition diagram

### Limitations

Importantly, every one of these regression models are descriptive in nature, and cannot speak to causal relationships between letter content and student or counselor characteristics. Our study focuses solely on potential inequities in the letter writing process. Although we suggest some implications for letter evaluation, our findings do not speak to the letter evaluation process itself, nor the role that letters play in actual admissions decisions. If granted data access, we hope to study the potential impact of letter differences on admissions probability in the future.

## Results

Table 4 displays in white the topics/outcomes discussed in this paper; topics displayed in gray are available upon request. The average number of sentences in each group is displayed in parentheses. Visual representations of the descriptive differences in letter content by key demographic traits are included in the online appendix. Tables 5, 6, 7, 8, 9, 10, and 11 report regression results for each outcome. Each column represents a different regression specification, with the exception of Model 1, where each *cell* is actually a different regression for each separate demographic variable. Each table shows how coefficient estimates change with each added set of controls when moving left-to-right across models. We begin by addressing key findings across school context, race/ethnicity, and SES, including findings related to students who scored in the 95th percentile on the SAT/ACT. We then discuss dynamics related to letter length, as well as topical outcomes of interest.

**Table 5:**
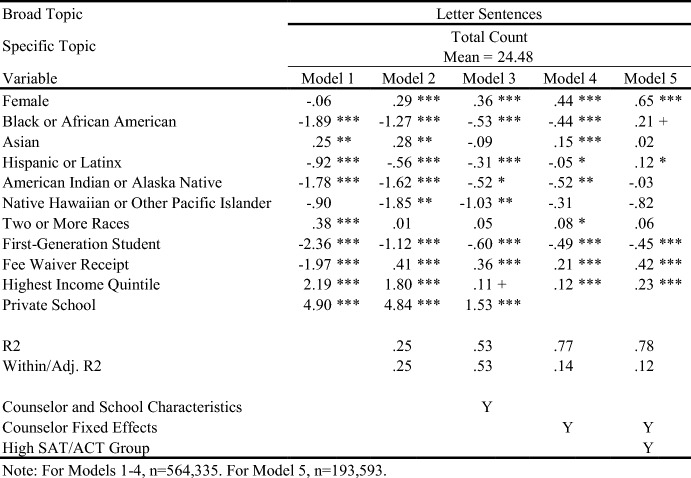
Regression results for total number of sentences in letters

**Table 6:**
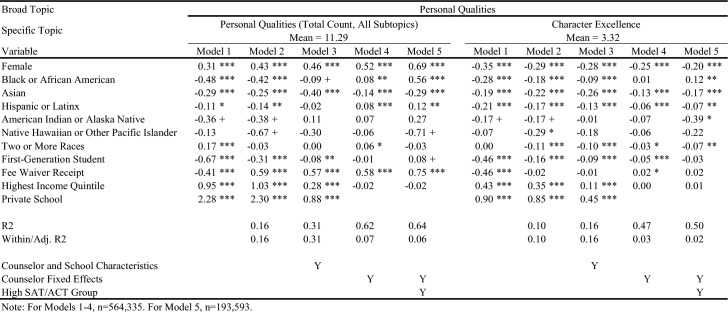
Regression results for letter sentences on personal qualities

**Table 7:**
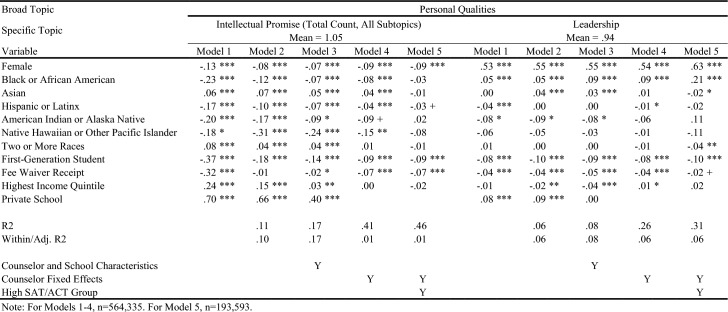
Regression results for letter sentences on personal qualities

**Table 8:**
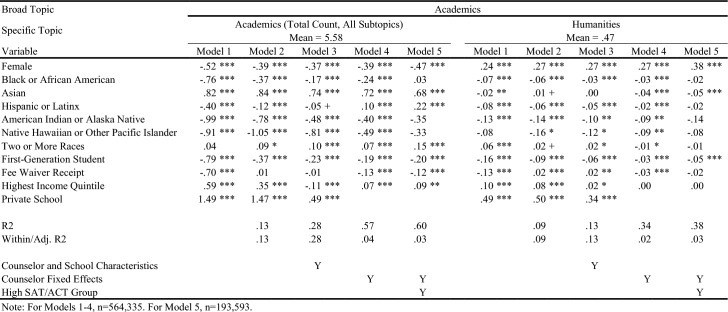
Regression results for letter sentences on academics

**Table 9:**
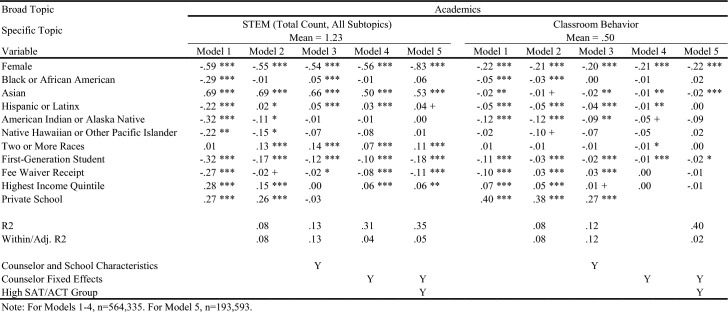
Regression results for letter sentences on academics

**Table 10:**
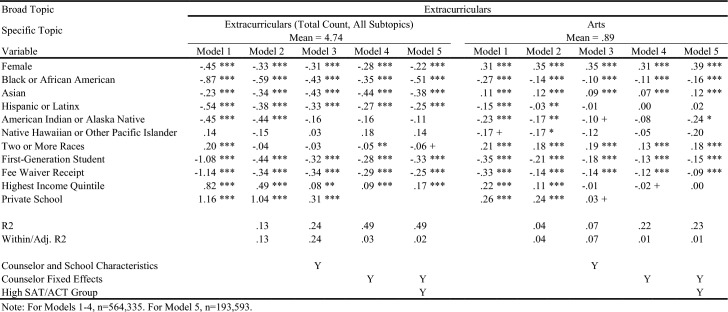
Regression results for letter sentences on extracurricular activities

**Table 11:**
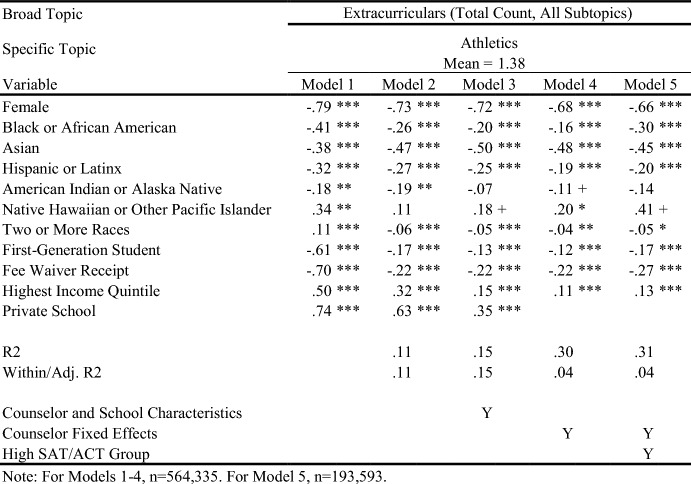
Regression results for letter sentences on extracurricular activities

### Variation in Length and Topic across School Context, Race/Ethnicity, and SES

When controlling for school type and demographic characteristics in Model 3, private school students had significantly more sentences overall (Table 5), as well as more sentences on Personal Qualities, Character Excellence, Intellectual Promise (Tables 6, 7), Humanities (Table 8), Extracurriculars, and Athletics (Tables 10, 11). Across tables, Model 4 shows results when comparing letters written by the same counselor (counselor fixed effects). Related to race/ethnicity in Model 4 across tables, Black and Latinx students relative to White students received letters with fewer sentences overall (Table 5), more sentences on Personal Qualities (Tables 6, 7), and fewer sentences on topics like Intellectual Promise, Humanities, Arts, and Athletics. Black, American Indian, and Native Hawaiian students received fewer sentences on Academic topics overall (Table 8). For SES in Model 4 across tables, first-generation students had letters with fewer sentences, and fewer sentences on Intellectual Promise, Leadership, Academics, Humanities, STEM, Extracurriculars, Arts, and Athletics. Similar patterns were apparent for students who received fee waivers, with two exceptions: These students received letters with more sentences (Table 5) and more sentences related to Personal Qualities (Table 6).

### Key Findings for Students with Higher SAT/ACT Scores

Some of the differences between groups identified in Model 4 were non-significant when controlling for test scores in Model 5. For example, Black students had letters with fewer sentences than White peers, even when comparing letters written by the same counselor (Table 5, Model 4). However, the difference was ameliorated when comparing students with similarly high test scores in Model 5. Similarly, Black students in general were less likely to have sentences on Intellectual Promise (Table 7, Model 4), but there was no significant difference when comparing students with the highest test scores. At the same time, some key disparities persisted among students with higher test scores, such as Black and Latinx students having significantly fewer sentences on Extracurriculars and Athletics (Tables 10, 11).

Even when comparing students with similarly high test scores, first-generation students received letters with significantly fewer sentences, while students from schools in the highest income quintile had letters with more sentences. Among high-scoring students, students who received fee waivers still had longer letters, and letters with more sentences on Personal Qualities, likely reflecting contextualization about their circumstances (e.g., overcoming adversity). In the next section, we will discuss how students who received fee waivers likely had longer letters at the expense of sentences about Academics or Extracurriculars; unlike private school students, who had longer letters but also significantly more sentences on Academics, Extracurriculars, and Athletics. Even when comparing only students with the highest SAT/ACT scores, fee waiver recipients and first-generation students had fewer sentences on Intellectual Promise (Table 7), Academics (Table 8), STEM (Table 9), Extracurriculars (Table 10), Arts (Table 10), and Athletics (Table 11).

### Dynamics Linked to Letter Length and Content

Now we unpack findings related to the number of sentences in letters (Table 5), while commenting on how topics discussed by counselors (Tables 6, 7, 8, 9, 10, and 11) may be linked to letter length. Table 5 exhibits the relationship between key variables and the total number of sentences in a letter. For students of color, the coefficient begins as being relatively large and meaningful, but progressively reduces in size through Model 5. This trend suggests that disparities in letter length by student race/ethnicity compared with White students are not driven by a single factor (e.g., student demographics, school/counselor characteristics, academic performance), but instead are related to all of the above. They are generally less prevalent among letters written for the highest scoring students by the same counselor. That said, if letters are not evaluated in that context (e.g., admissions readers are not trying to “norm” their interpretation of a letter by comparing it against other letters written by the same counselor – which is quite possible), the racial disparities observed in Models 1 and 2 may remain issues of concern. The coefficients on female and fee waiver recipients reflect an opposite trend: They start negative or insignificant in Model 1 and progressively grow by Model 5. This indicates that these students are actually receiving slightly longer letters the more we account for school/counselor and academic characteristics, compared with male and non-fee waiver recipients.

Private school students seem to have letters roughly 20% longer than public school students (using the sample mean as a benchmark), even accounting for other student demographics and socioeconomics in Model 2. This aligns with the hypothesis that greater resources and reduced time constraints on counselors in the private school context may advantage these students.

As described earlier, differences in overall letter length can be thought of as the sum of demographic differences across all broad categories of topics combined. We can examine whether certain topics of discussion are driving differences in letter length more than others by looking at Tables 6, 7, 8, 9, 10, and 11. For example, the coefficient for fee waiver recipients for Personal Qualities sentences (Table 6) is larger than the corresponding coefficient for overall sentences (Table 5) across all models. This indicates that while fee waiver recipients receive longer letters than non-recipients after controlling for other demographic characteristics, they specifically have more sentences in their letters about Personal Qualities. However, because these coefficients are larger, they are also receiving fewer sentences about Extracurriculars, Academics, or Other topics. Likewise, about half of the difference in overall sentences we observed for private school students versus public school students seems to be driven by private school students having more Personal Qualities sentences. In terms of race/ethnicity, Black/African American and Hispanic/Latinx students are generally receiving fewer sentences about Personal Qualities up until the high achieving subsample, in which they receive 0.557 and 0.121 more sentences, respectively, about Personal Qualities than White students. Asian/Asian American students across all specifications received slightly fewer sentences about Personal Qualities than White students.

For Academics (Table 8), we see a roughly inverse trend: Groups that had positive coefficients for Personal Qualities have generally negative coefficients for Academics, except for higher income and private school students. For example, female and fee waiver recipients generally have fewer sentences about Academics across all models, while Asian/Asian American students generally have far more sentences about Academics across all models. These findings suggest that the Personal Qualities sentences are in effect “crowding out” sentences about Academics when counselors write their letters for certain groups. (This dynamic seems less applicable to Latinx students,where coefficients shift from negative to positive across models for both topics.) This “crowding out” dynamic is true for most groups except the highest income quintile and private school students, where the coefficients for both Personal Qualities and Academics remains positive across most models, as shown in Tables 6 and 8. Rather than crowding one another out, the letters for private school students and those from wealthy areas are simply longer overall. Reflecting this dynamic, we see such large, positive coefficients for these groups in the overall sentence count regressions. Table 10 displays results for sentences about the broad topic category of Extracurriculars. Results here are far more uniform. Female, lower-SES, and URM students all see fewer sentences about Extracurriculars across all models. Both high income and private school students have generally positive coefficients across all Extracurricular models.

### Detailed Topics within Personal Qualities, Academics, and Extracurricular Activities

Turning to findings on more detailed topics, in Tables 6 and 7, we examine sentences related to Character Excellence, Intellectual Promise, and Leadership. Female students received relatively fewer sentences about both Character Excellence and Intellectual Promise than Male students, but instead got *substantially* more sentences about Leadership – a difference of between 50–60% relative to the mean across models. Importantly, these Leadership sentences are ones about leadership in general, whereas leadership in Athletics or specific Extracurriculars would likely instead be classified into those other topic areas. Fee waiver recipients and first-generation students tend to have negative coefficients for all three topics, across all models, while private school and the highest income quintile students tend to have positive coefficients. Trends by race/ethnicity are more mixed – Black/African American students have negative coefficients for Character Excellence in Models 1–3, but a positive coefficient in Model 5. They have uniformly negative coefficients for Intellectual Promise, but then uniformly positive coefficients for Leadership. Hispanic or Latinx students have slightly negative coefficients across all three topics, for all measures. Asian/Asian American students have fairly substantially negative coefficients across all models for Character Excellence, but positive coefficients for all models in Intellectual Promise except Model 5 for the high achieving subsample.

In Tables 8 and 9, we can examine detailed topics within Academics: Humanities, STEM, and Classroom Behavior-related sentences. Black/African American students have negative and insignificant coefficients across all three topics, while Asian/Asian American students have weakly negative coefficients for Humanities and Classroom Behavior, but extremely positive coefficients for STEM. First-generation and fee waiver students have negative coefficients consistently across all three topics, while private school students have highly positive coefficients across all three. In fact, the relative difference for private school students for Humanities is at times greater than 100% more than the mean, and is quite large for Classroom Behavior at between 55 and 80% of the mean. Lastly, trends for Extracurricular topics (Tables 10 and 11) parallel findings related to Academics. First-generation and fee waiver recipients have negative coefficients in all models across both Arts and Athletics, while highest income quintile and private school students have positive coefficients in nearly all models across both. All racial/ethnic minority students have fairly consistent negative coefficients across all models for both topics, with the exception of Asian/Asian American and Two or More Races students for Arts, where coefficients are generally positive.

## Discussion

Several findings stand out. First, overall letter length varies notably by economic background, and to some extent, race/ethnicity. First-generation students received shorter letters than continuing-generation students across numerous topics. Their letters were shorter even conditional on demographic traits, school/counselor characteristics, counselor fixed effects, or having high test scores. Fee waiver recipients generally received longer letters than their non-recipient peers across models, but their letters were generally more focused on Personal Qualities topics *at the cost* of sentences about Academics or Extracurriculars—perhaps due to counselors using letters to explain home life and financial circumstances for lower-income students.

Nearly all groups besides Asian/Asian American students had shorter letters than White students across most models, although differences were ameliorated when only examining students with the highest test scores. Counselors may be inclined to provide additional detail about students when they have higher test scores. Findings suggest that letter inequities related to race may be most obvious or concentrated in letters written for students outside the high test-taking subsample (i.e., the vast majority of test-takers), and further reinforce that these inequities are closely related: Inequities in standardized testing have repercussions for inequities in letters.

Private school students and students living in higher-income communities generally had longer letters. Private school students had significantly more sentences on Personal Qualities, and unlike low-income students, these sentences did not “crowd out” content on other topics like Academics. Private school students also gyunenerally had more sentences about Character Excellence, Intellectual Promise, and Leadership—topics that generally had negative coefficients for first-generation and fee waiver recipients. For private school students and students from high-income communities, longer letters included more sentences about Extracurriculars and Athletics. Of note, private school students had more sentences on Humanities (100% more than the mean), Classroom Behavior, and in general, STEM—once again, topics that received less coverage in letters for first-generation students and fee waiver recipients. Longer letters and differences in topical content may reflect the benefits inequitably distributed to students attending schools with lower student-to-counselor ratios, with counselors knowledgeable on what content to include, as well as greater bandwidth and resources to write letters (Chetty et al., 2023; Schwarz, 2016).

Returning to race/ethnicity, all student race/ethnicity groups had fewer sentences about extracurriculars than White students across all models. These findings reflect scholarship on the inequities associated with extracurricularinvolvement (Park et al., 2023). Asian/Asian American students had consistently fewer sentences about Personal Qualities and consistently more sentences about Academics across all models. Asian American students had slightly fewer sentences noting Character Excellence in letters across models.

We cannot speak to the role these various differences and disparities may play in the ultimate evaluation of a student’s application portfolio. Regardless, findings add to our understanding of *how* privilege and inequity influence admissions through multiple pathways. Privileged extracurricular activities and longer, more personalized letters of recommendation may be favored by admissions readers; both are more common among private school students (Chetty et al., 2023; Schwarz, 2016). Our findings show how these areas can reinforce one another. For example, letters for more affluent and private school students are more likely to comment on activities like Athletics, which may contribute to more positively valued letters. Our findings confirm at large-scale some of the conclusions drawn from closer qualitative analysis of letters from private school counselors (Schwarz, 2016), which note the multiple advantages that these students receive through letters of recommendation.

Our work also adds to understanding of how Personal Qualities are discussed in letters, and how groups may benefit differently from such discussions. As noted, we found that private school students had letters with more sentences featuring Personal Qualities, and generally, such sentences did not come at the expense of discussion of other topics—instead, applicants just had longer letters. Fee waiver recipients also had letters with more sentences on Personal Qualities, but such commentary “crowded out” discussion of other topics. Likely for these students, discussion of Personal Qualities includes insight related to the degree that an applicant has experienced and overcome challenges, which may benefit applicants even if overall letter quality is lower in some regards (Rothstein, 2022). Based on analysis of letters at UC Berkeley, Rothstein (2022) noted, “There is a case for including subjective information like letters in the process in order to make it more visible, at least within systems like Berkeley’s that are carefully designed to promote equitable admissions” (p. 13). Rothstein’s comments suggest that letters are most beneficial when the admissions process is highly calibrated towards promoting equity, versus a system where letters are read without adequate consideration to the structural factors affecting applicant opportunity shaping such letters. Overall, letters can work as a sort of double-edged sword, where (if left unchecked) they can perpetuate privilege for some, while having the potential to disrupt and contextualize inequity for others.

## Conclusion

We add to the body of work indicating that, like other components of college applications, letters of recommendation from high school counselors are subject to numerous inequities. Findings related to private school students (e.g., longer letters, more sentences on Personal Qualities, Athletics, Intellectual Promise, Character Excellence, Humanities, etc.) speak to how elite admissions privileges this population through prioritization of “personal” factors gleaned from parts of the application like letters of recommendation and extracurricular activities (Chetty et al., 2023; Rosinger et al., 2021). At the same time, discussion of personal characteristics for historically underrepresented populations can reveal important contextual information regarding a student’s experiences overcoming adversity or dealing with other circumstances (Rothstein, 2022) that might improve equity in holistic admissions practices (Bastedo & Bowman, 2017).

Should institutions keep letter requirements or not? Based on our own work and the work of others (Rothstein, 2022), there does not appear to be a “slam dunk” answer. Our findings offer insight and considerations for both those who support and oppose letter requirements. Some schools have already chosen to not require letters (University of Illinois, Urbana-Champaign), and others request them only for some students, like institutions in the University of California system. On the whole, discarding letter requirements or only asking certain students to submit letters when additional context about the students is needed, are viable options. Beyond inequities affecting letters that we have documented, letters can pose a considerable burden to teacher/counselor workload. These two dynamics provide some support for doing away with letter requirements or reducing them (i.e., only requesting them for certain students), which may be attractive to large state systems.

At the same time, the field would benefit from additional research gauging how admissions decisions are affected by the inclusion or exclusion of letters of recommendation, and understanding of how different groups benefit (or do not benefit) from inclusion or exclusion of letters. Researchers could conduct randomized control trials, ethnographic work, and other types of research to examine how decisions are shaped (if at all) by the inclusion of letters under different conditions. Such studies would be beneficial both at the national level, as well as the institution-specific level. Researchers could examine any impact associated with letters with similar content, but different lengths, or how discussions among reviewers are affected when letters are included or not included in the portfolio. They might examine whether and how different combinations of admissions materials (e.g., one teacher letter versus two teacher letters, a counselor letter but no teacher letters, teacher letters but no counselor letter, etc.) shape decision-making processes. Additional inquiry is also needed to understand the potential impact of artificial intelligence on letters and how the increased usage of such tools will affect the field, for better or for worse.

For institutions that *do* want to keep letters, our findings demonstrate how it is critical that admissions officers read letters in the context of structural opportunity, with special consideration to information that helps contextualize applicants from historically underrepresented backgrounds. Ultimately, implications of differences in letters between groups for equity hinges on exactly *how* letters are contextualized and normed in evaluation processes. Keeping letters of recommendation will be most beneficial in systems where holistic review practices are calibrated to prioritize equity and not overly reward privilege. Likely, private institutions are most inclined to want to keep letter requirements, although institutions and application platforms should consider ways that they could lessen the workload on teachers and counselors. These efforts could include moving to a more standardized length of letters across counselors and high school contexts as one way to reduce the workload for school counselors, teachers, and admissions staff. Such a change could lessen potential positive bias towards students who simply have longer letters.

Institutions also should provide sustained training on the ways in which bias, inequity, and school resources can influence letters (McDonough, 1997; Schwarz, 2016). Given that few admissions professionals report being prepared to consider a student’s context for opportunity when reviewing applications (Lee et al., 2022), institutions must train readers on the contexts that shape letters. At a broader level, continuing to diversify high school counselors, as well as admissions staff, is also important. Both groups are known for their homogeneity (ASCA, 2023), which may influence both the writing and reading/scoring of evaluations (Bowman & Bastedo, 2018; Linnehan et al., 2011).

Some have argued that inequities affecting letters of recommendation are a reason for why institutions should bring back standardized tests (see n.a., 2024). However, existing research, including the present study, does not necessarily support that argument. As noted, the field would benefit from additional research on the subject, such as how admissions decisions are affected depending on varying conditions (e.g., letters with test scores, letters without test scores, letters reviewed only for low-income students with or without test scores, etc.). Regardless of whether institutions bring back required testing or not, those that continue to require letters must remember to read them in the context of structural opportunity.

Given that the Supreme Court ruling limits how institutions may consider race/ethnicity in admissions decisions, but does not prevent them from “considering an applicant’s discussion of how race affected his or her life,” (*SFFA v. Harvard*, 2023, p. 39), institutions must continue work to identify, recruit, and admit talented students from all backgrounds. However, admissions readers are increasingly missing critical information to help them robustly contextualize student applications. Institutions must invest heavily in encouraging applications from students from historically excluded and underrepresented backgrounds, work to broaden access, recalibrate admissions systems to address inequality, and work to improve equitable, mission-driven practices. Holistic review processes must be leveraged to contextualize inequality in letters and other non-standardized components of the application, facilitating admissions decisions that identify talented students from a wide range of backgrounds.

## Supplementary Information

Below is the link to the electronic supplementary material.

Electronic supplementary material 1 (DOCX 1319 kb)

## Footnotes

1 The overwhelming majority of our sample applicants from this season (> 99%) had already submitted their application prior to the onset of the pandemic in February 2020.

2 Those familiar with the Common App recommendation process also know that counselors can optionally rate a student’s character across a variety of measures (e.g., “Maturity,” “Leadership”, “Academic Potential,” and so on) on a likert scale. As these are not required and thus not systematically complete for all students, we do not currently rely on these data in our present analysis. We hope to analyze these data more directly in future work.

3 We rely on the Common App’s currently operationalized definition for domestic applicants: students who are either U.S. Citizens (living domestically or abroad, and whether sole or dual-citizens) or otherwise permanent residents of the U.S. (whether documented or undocumented). In other words, we exclude students who claim sole citizenship to a country besides the U.S.

4 We define substantive length in this context as containing at least 4 sentences of at least 20 characters each after the text cleaning processes are completed. These thresholds were set somewhat arbitrarily based on the ad hoc reviews of letters above and below these thresholds; those below the threshold were overwhelmingly erroneous (e.g., composed entirely of gibberish text due to the PDF reading process) or unanalyzable (e.g., a counselor submitting only the single sentence “I do not know this student adequately enough to evaluate them” despite not checking the option to opt-out of writing a letter entirely).

5 Though it is the case that applicants may apply across multiple seasons, we include only the most recent application we observe from a given applicant in our sample. Internal analyses at Common App show that students we observe multiple times are often first Juniors testing their options before a “serious” application season their Senior year, in which case their Senior year data are most updated and complete.

6 Those who inspect our online codebase will see that minor alterations were necessary in the case of the topic modeling and sentiment analysis procedures only to account for the vastly larger data size – handling these data required a batch-analysis approach not necessary for the development sample. These adjustments were manually verified to cause no changes to the output of either analysis.

7 Note that in this strategy, we may split *high schools* across development and analysis subsamples in circumstances where there are multiple letter-writing counselors in a given school. We ultimately choose not to use high school fixed effects as high schools are generally fixed within counselors, and so it is largely duplicative of counselor fixed effects; as such, splitting high schools across subsamples is not a concern for our analysis.

8 As the Common App only includes four-year institutions, our definition of first-generation implies that students reported that no parent completed any four-year degree – whether in the United States or outside of it.

9 Applicants self-identify as eligible for the fee waiver, and eligibility criteria include common indicators like receipt of an SAT/ACT test fee waiver, receipt of free or reduced price lunch, receipt of public assistance, participation in a low-income student program like TRIO, and so on.

10 We use the conventions employed by the National Science Foundation: applicants identifying as Black or African American, Latinx, Native American or Alaska Native, or Native Hawaiian or Other Pacific Islander are classified as URM applicants.

11 We removed obviously erroneous values (e.g., scaled GPAs higher than 1.5 and lower than 0.5) given the likelihood of reporting issues in these instances, though allow for values higher than 1.0 given the prevalence of weighted GPA schemes.

12 Note that the 2018 and 2019 columns do not sum to the pooled column for the number of counselors and schools due to the fact that many counselors and schools appear in both years of the data. This is intuitive if counselor turnover is relatively low from year-to-year and a given school consistently has students applying to college through the Common App from year-to-year as well.

13 This differs from the student-level statistic for letter length because it is calculated at the counselor-level, which weights observations differently. That this statistic is smaller than the student-level statistic likely indicates that, in general, counselors who wrote *more* letters actually wrote *longer* letters (i.e., when letter length is averaged at the student level, the many longer letters written by fewer counselors tip the average up; when collapsed to the counselor-level, they are weighted relatively lighter and thus the average is tipped back down).

14 We rely on this platform-based proxy because it is available for *all* schools, whereas more standard student-counselor ratios tend to only be available for public schools in national datasets; this would thus make unusable nearly a quarter of all letters in our data for ensuing analysis.

15 We focus only on the past two years because the data to track counselor identities and forms change systematically when looking earlier than the 2016-2017 application season. As such, we can’t currently look more than two years prior to the 2018-2019 season without undergoing substantial additional data cleaning.

16 While sentiment analysis is another attractive NLP technique to apply to these evaluative letters, prior work by Kim (2022) found that negative sentences are exceptionally rare in the parallel teacher recommendation context. Moreover, sentiment analysis cannot adequately explain when a negative (or neutral) sentence is actually *beneficial* to a student. For example, a sentence describing a student’s difficult financial circumstances would likely be read by the algorithm as negative, but nonetheless this sentence might serve to support the student’s consideration by admissions officials. Similarly, a sentence about a student’s transferring of schools would likely be read as neutral by the algorithm, but again could be beneficial for the student’s consideration by admissions officials. The ambiguity of whether more neutral or negative sentences (and thus fewer positive sentences) is thus conceptually muddy enough that we opted not to include these sentiment analyses here for clarity and concision.

17 We leverage the SentenceTransformers library to conduct this step of the analysis, known more formally as constructing Sentence Embeddings. Our main model uses the “all-mpnet-base-v2” pre-trained model derived from Microsoft’s work, though we also tested the “all-distilroberta-v1” pre-trained model derived from HuggingFace’s work.

18 Importantly, because BERTopic is able to create extremely granular and even idiosyncratic topic clusters, we group substantively related clusters together into a “supertopic” to maximize interpretability. All references to “topics” in this paper are referring to these aggregated supertopics.

19 BERTopic’s clustering algorithm also allows for sentences to not be assigned a topic at all, which can present a threat to our analysis somewhat similar in nature to data being missing-not-at-random. That being said, we find that an overwhelming minority of sentences fall into this category (~ 2.5% of all sentences analyzed, or an average of half a sentence per student). While systematic missingness could bias our results, the magnitudes here are such that all of the main findings we highlight in the narrative would not change even in the worst case scenario of “perfectly biased” differential missingness.
