# Supplementary material for: Letters of Recommendation by High School Counselors in Selective College Admissions: Differences by Race and Socioeconomic Status in Letter Length and Topics Discussed
Source: Res High Educ. 2025 Jul 4;66(5):30. doi: 10.1007/s11162-025-09847-5 (PMC12227477; doi:10.1007/s11162-025-09847-5)

Online Appendix

In this appendix, we address the issues of letter submission type (i.e., upload with PDF conversion versus open-text entry) as well as inter-rater reliability. We also provide visual representations of the descriptive differences in letter content by key demographic traits. Related to letter submission type, these two separate submission types result in us receiving vastly different types of de-identified text data (Common App uses the proprietary Amazon Comprehend service to detect and remove personally identifiable information (addresses, phone numbers, names, etc.) from all text before it is received by analysts; the PDF letters must be analyzed through an optical character recognition algorithm to turn the PDFs back into analyzable text, whereas the open-text response field does not. Counselors are also far more likely to include additional text like school letterhead, school addresses, dates, and so on, in the PDF format than in the open text response field. Finally, the open-text response field enforces a 1000 word limit on length, whereas the PDF letters do not. As a result, these two formats require substantially distinct text cleaning operations to filter down to the “real” content of the letter for us to analyze, and may not even necessarily be comparable. This motivates our desire to focus on PDF letters – we obtain the vast majority of the sample of letters and are able to ensure the consistency of text cleaning for them, rather than attempting to compare across two very distinct data-generating processes.

It is also important to note that open-text letters tend to be more commonly submitted for students attending public schools (90% of open-text letters are for students attending public schools, while 74% of PDF letters are), lower-income students (32% of open-text letters are for students receiving a fee waiver, while 22% of PDF letters are; 39% of open-text letters are for students living in top quintile ZIP codes, while 65% of PDF letters are), and first-generation (31% versus 21%). Nonetheless, it remains the case that the overwhelming majority of letters submitted by public school counselors are submitted in PDF letter format. That being said, our exclusion of the open-text letters may result in our analyses producing *underestimates* of the disparities we measure, if the open-text letters we exclude are generally written by lower-resourced counselors and are shorter due to the word limit.

Next we address inter-rater reliability (IRR). With the “common” sentences, we first moved to measure IRR among the human team with Light’s Kappa (essentially, the average level of agreement across each pairwise set of raters; Hallgren, 2012). Given that reasonable people can disagree about the primary topic of any one sentence, this human-only IRR value would establish a realistic baseline for how to appraise the algorithm’s performance – put another way, 100% agreement with humans is an unrealistic target for the algorithm if humans cannot achieve that level of agreement with one another. We can then see how these IRR values change when we add in the actual algorithm’s output; a meaningful decrease in the IRR once the algorithm is added would suggest it disagrees with the human raters more than the human raters disagreed with one another, while no change would suggest it disagrees with the human raters about as often as the human raters disagreed with one another.

We can moreover benchmark the algorithm’s actual performance against a series of hypothetical scenarios to serve as additional points of comparison for IRR performance: one in which the algorithm just randomly guesses a random topic from the set of possible topics, one in which the algorithm provides a random guess pulled from the distribution of human ratings, one in which the algorithm “cheats” by selecting the topic that would result in the *lowest* level of agreement with the human raters (what we can think of as the hypothetical floor for IRR with the algorithm), and one in which the algorithm “cheats” by selecting the topic that would result in the *highest* level of agreement with the human raters (what we can think of as the hypothetical ceiling for IRR with the algorithm).

In Figure A1, we find that the algorithm generally agrees with humans at roughly the same level that humans agree with one another: the human-only IRR was 0.528, while the IRR with the algorithm was only slightly lower at 0.516. For context, the hypothetical lowest IRR possible was 0.368 (“adversarial guessing”), while the highest possible was 0.574 (“complementary guessing”). This shows that while it was *hypothetically* possible for the algorithm to perform better, its performance is quite close to as good as we could have hoped in comparison to other human raters. For those who may be concerned at the overall level of IRR here (some researchers suggest a threshold of 0.6 or higher for strong reliability), we can also simplify our topic assignments instead to a broader category of topic: Academics, Extracurriculars, Other, and Personal Qualities. Thus, we are making less specific arguments about what a sentence is about, but are doing so with greater reliability. Figure A2 displays the results of this exercise, revealing as expected that the humans-only IRR rises substantially to 0.658 (from 0.528), and the IRR rises to 0.633 with the algorithm included (from 0.516). The theoretical upper bound for IRR here is 0.695, while the theoretical lower bound is 0.419. Note here that the IRRs of random and distributional guessing rise as well – given that there are so many fewer options to guess from, this is an expected mechanical relationship.

Importantly, we stratified the random sample of sentences being examined by humans specifically to also assess the extent to which the algorithm may exhibit a degree of algorithmic bias; that is, might the algorithm perform better for students of one demographic over another? In Figure A3, we find that this is not the case for student sex or public/private school status, and, interestingly, also that our IRR with the algorithm is meaningfully higher for URM students versus non-URM students. That being said, we actually see that this pattern exists even among only our human coders, as shown in Figure A4. Therefore, it may be the case that the addition of the algorithm does not exacerbate this issue in any meaningful way. In other words, the demographic IRR issues shown in Figure 5 seem to be driven as much by the human coders as it is by the algorithm. This could be an artifact of the 100 sentences we ultimately sampled, in that there just happened to be more ambiguity in the sentences from non-URM students by chance, resulting in “true” grounds for disagreement, or due to systematically different styles of writing when counselors write about non-URM students that produces greater ambiguity or complexity in interpretation. This seems unlikely to be driven by biases present in our human readers, as readers had no access to student demographic information throughout this process (besides student pronouns used in the sentences). Further, we manually verified that there were no obvious clues about student race/ethnicity in the sentences themselves.

### Figure A1. Inter-rater Reliability Statistics across Varying Group Scenarios, All Topics


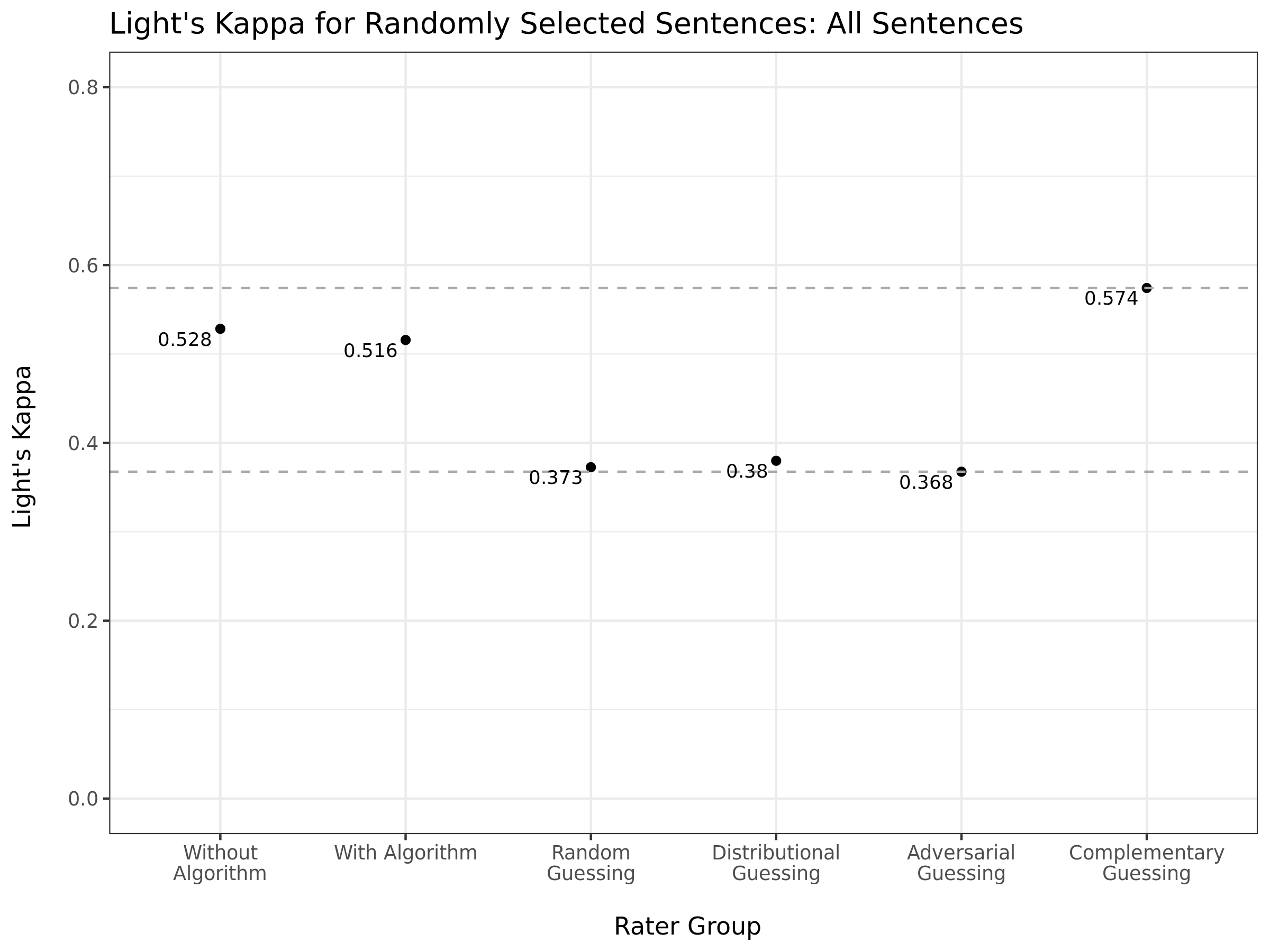


### Figure A2. Inter-rater Reliability Statistics across Varying Group Scenarios, Broad Topic Categories


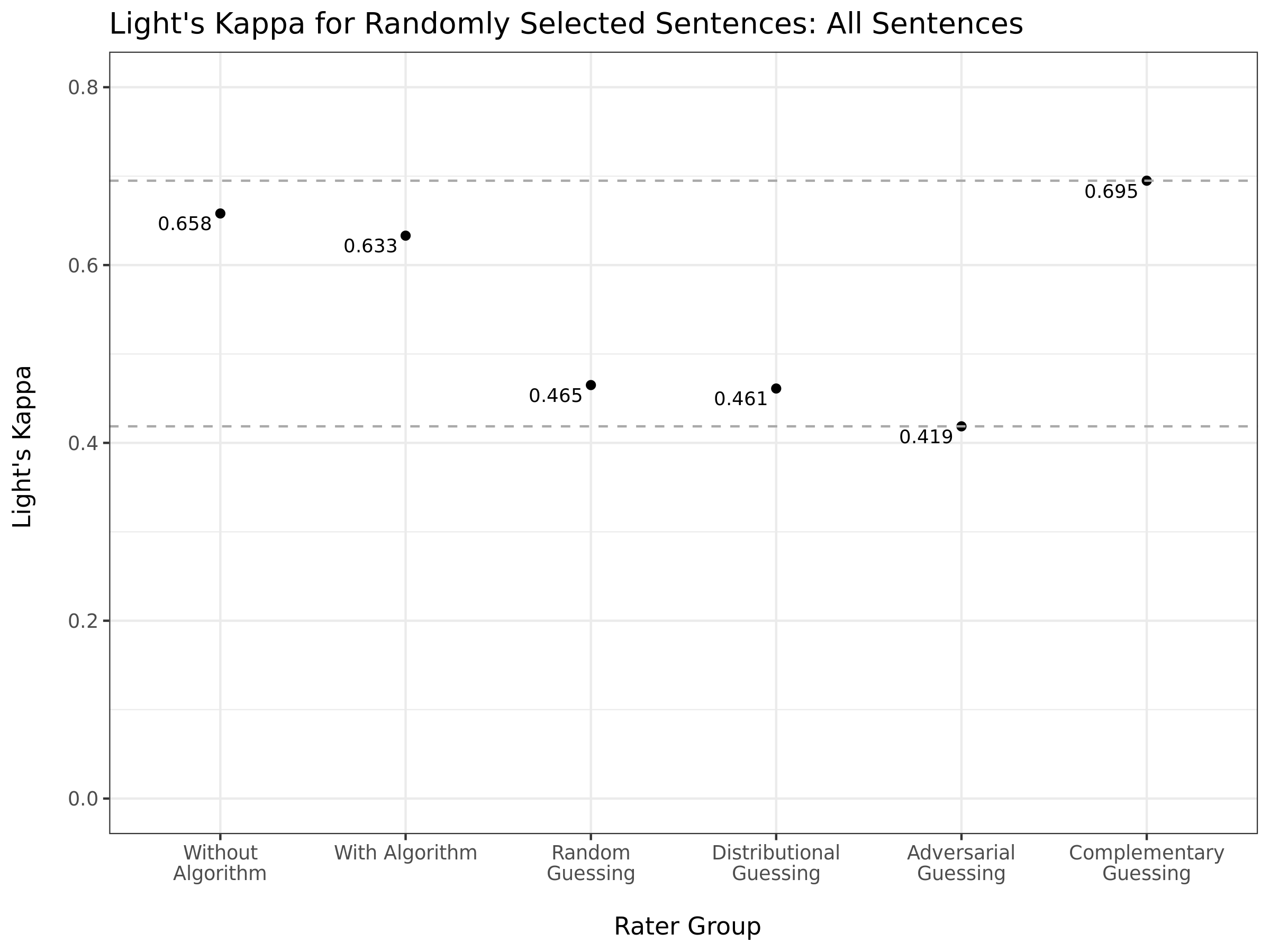


### Figure A3. Inter-rater Reliability Statistics across Student Demographics, With Algorithm


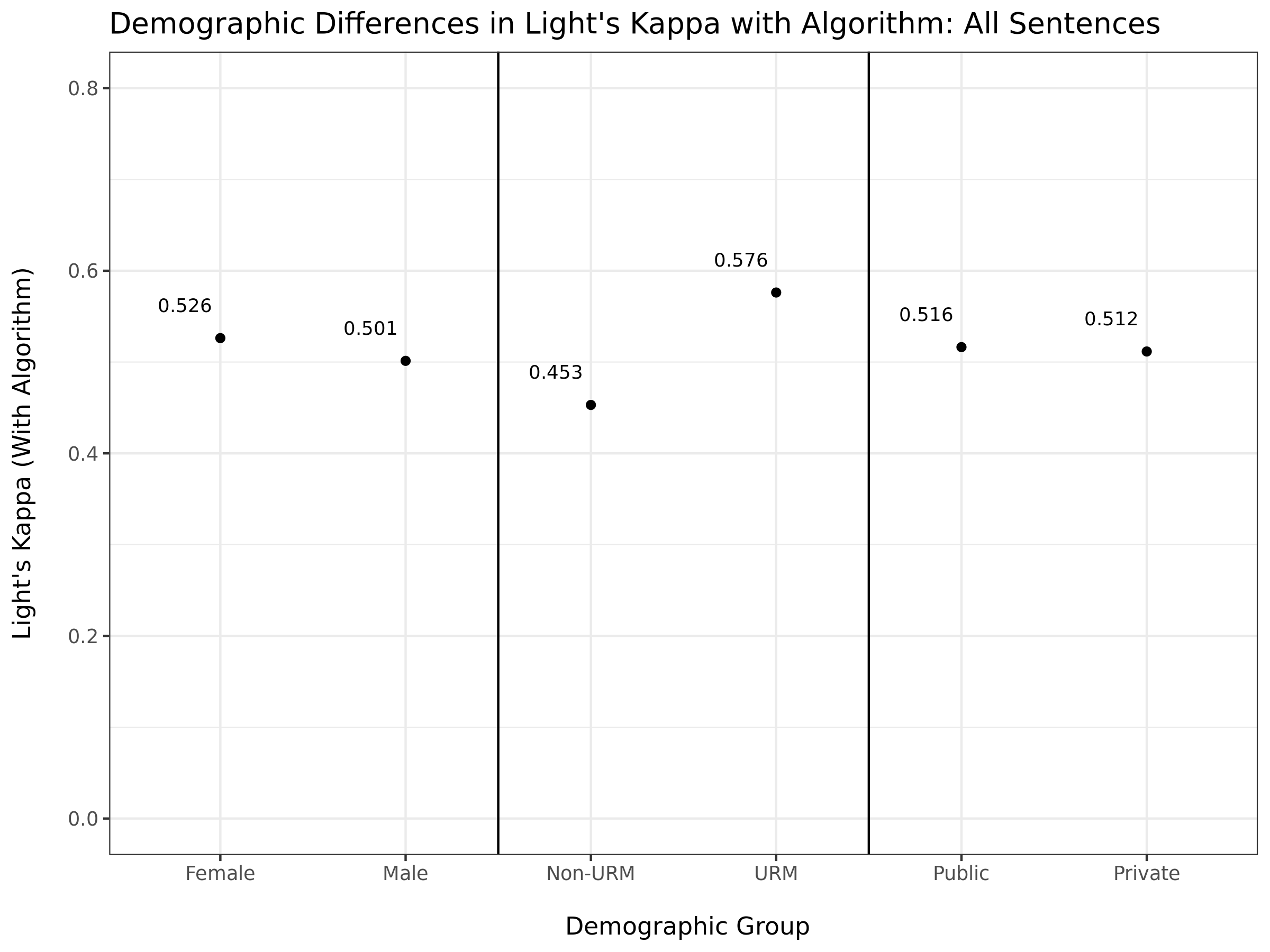


### Figure A4. Inter-rater Reliability Statistics across Student Demographics, Without Algorithm


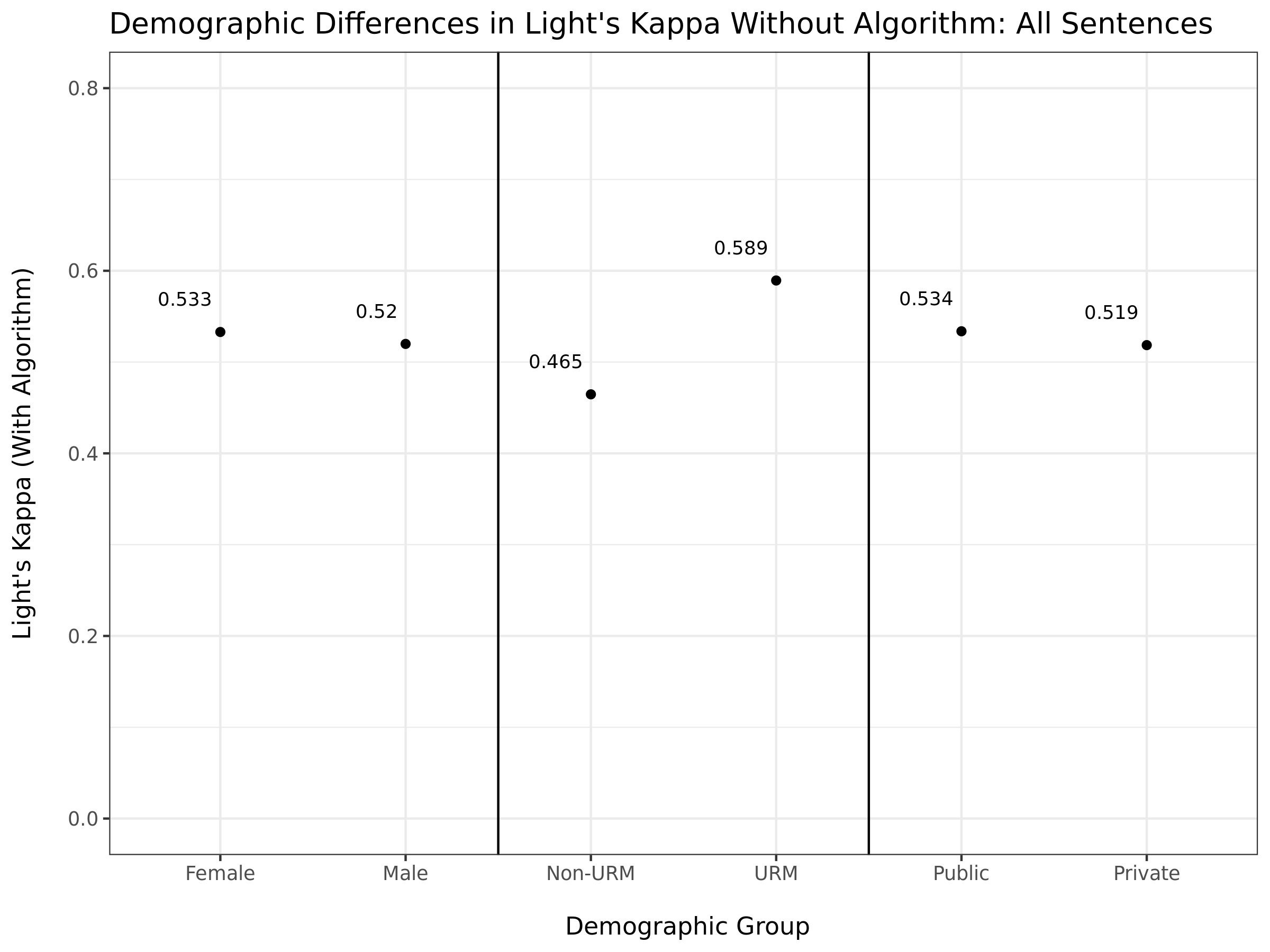


###

### Figure A5. Descriptive Differences in Letter Content by URM Status

Differences relative to sample mean (displayed in parentheses on Y-axis)


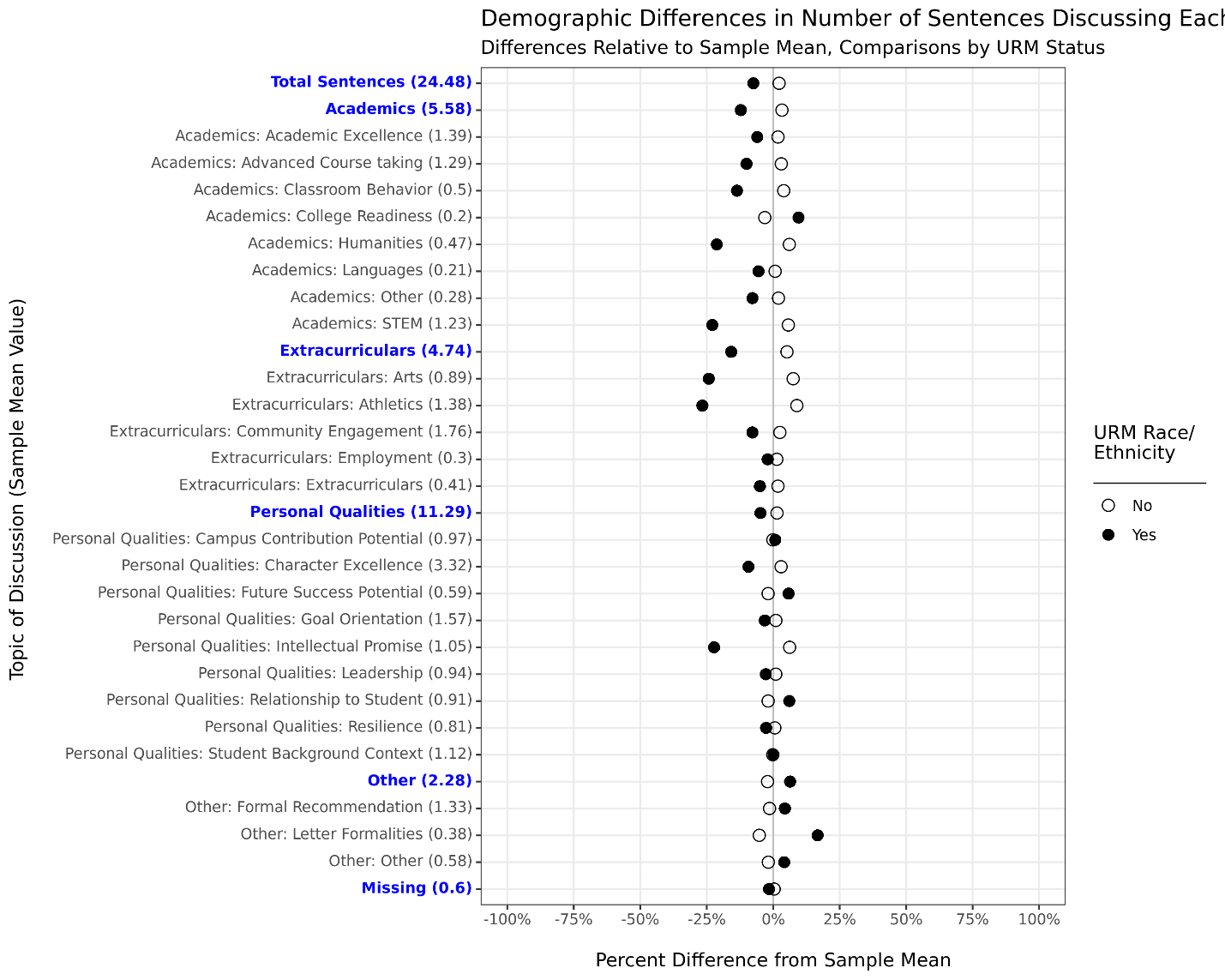


###

### Figure A6. Descriptive Differences in Letter Content by First-Generation Status

Differences relative to sample mean (displayed in parentheses on Y-axis)


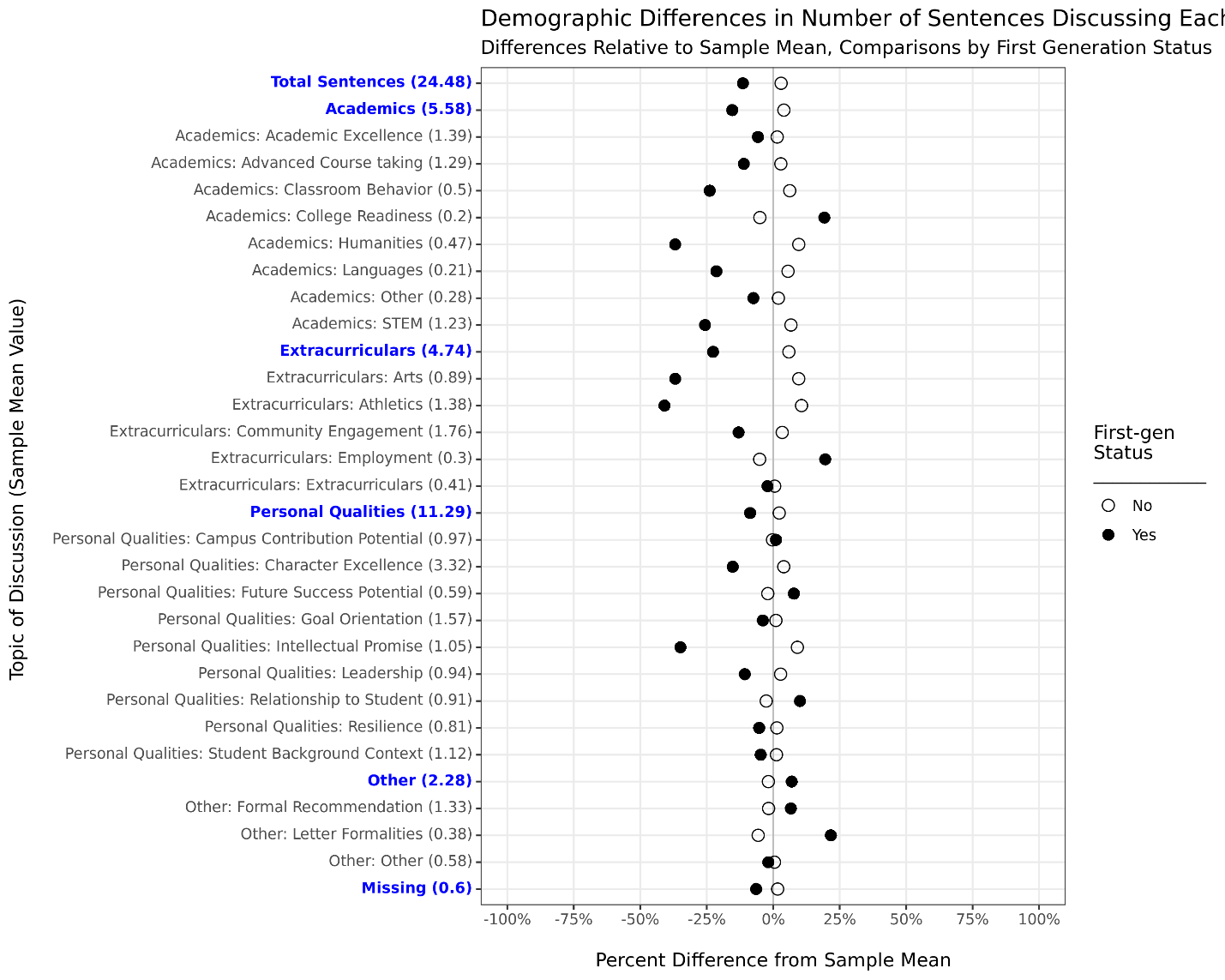


###

### Figure A7. Descriptive Differences in Letter Content by Fee Waiver Eligibility

Differences relative to sample mean (displayed in parentheses on Y-axis)


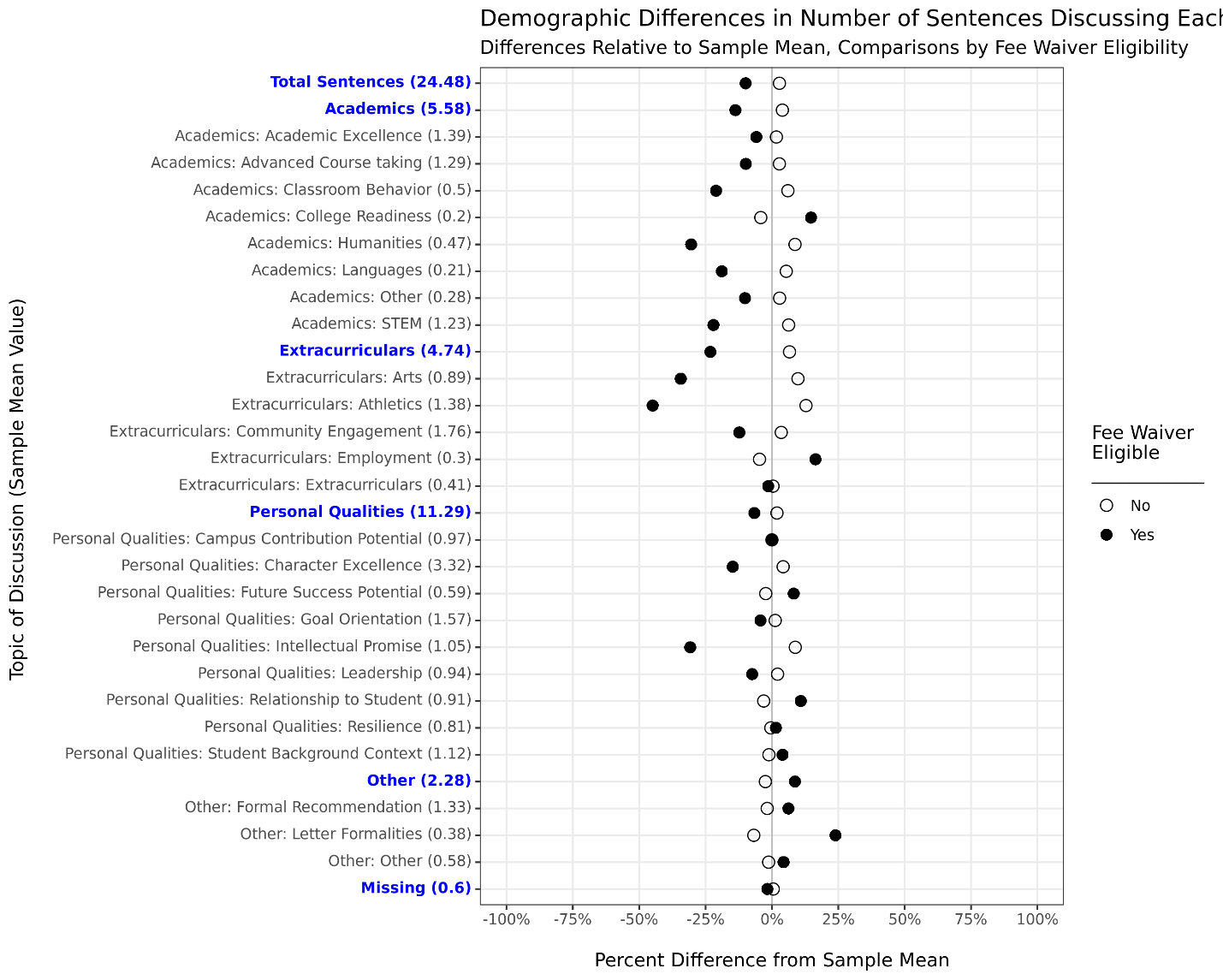


###

### Figure A8. Descriptive Differences in Letter Content by ZIP-Code Income Quintile

Differences relative to sample mean (displayed in parentheses on Y-axis)


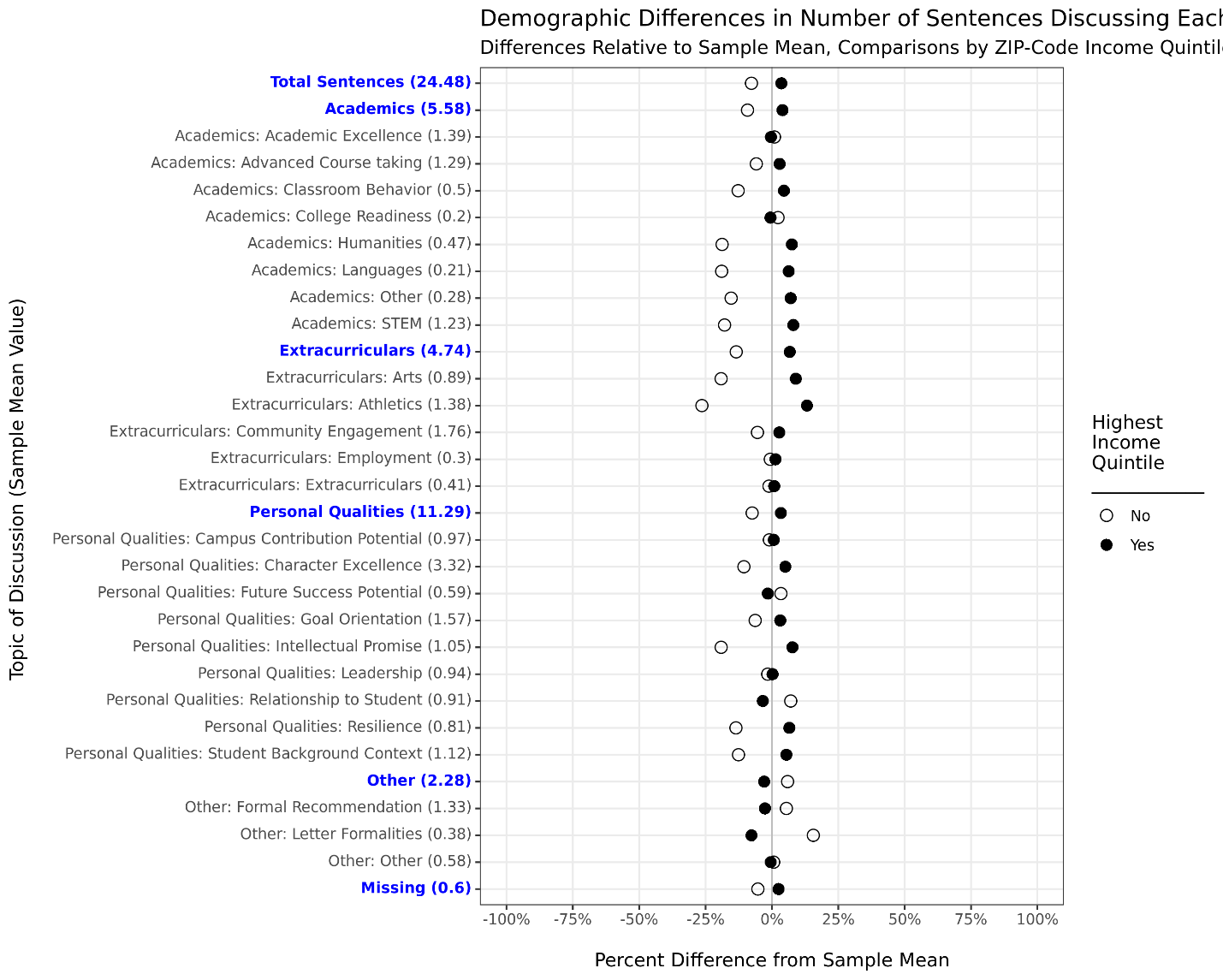


###

### Figure A9. Descriptive Differences in Letter Content by School Sector

Differences relative to sample mean (displayed in parentheses on Y-axis)


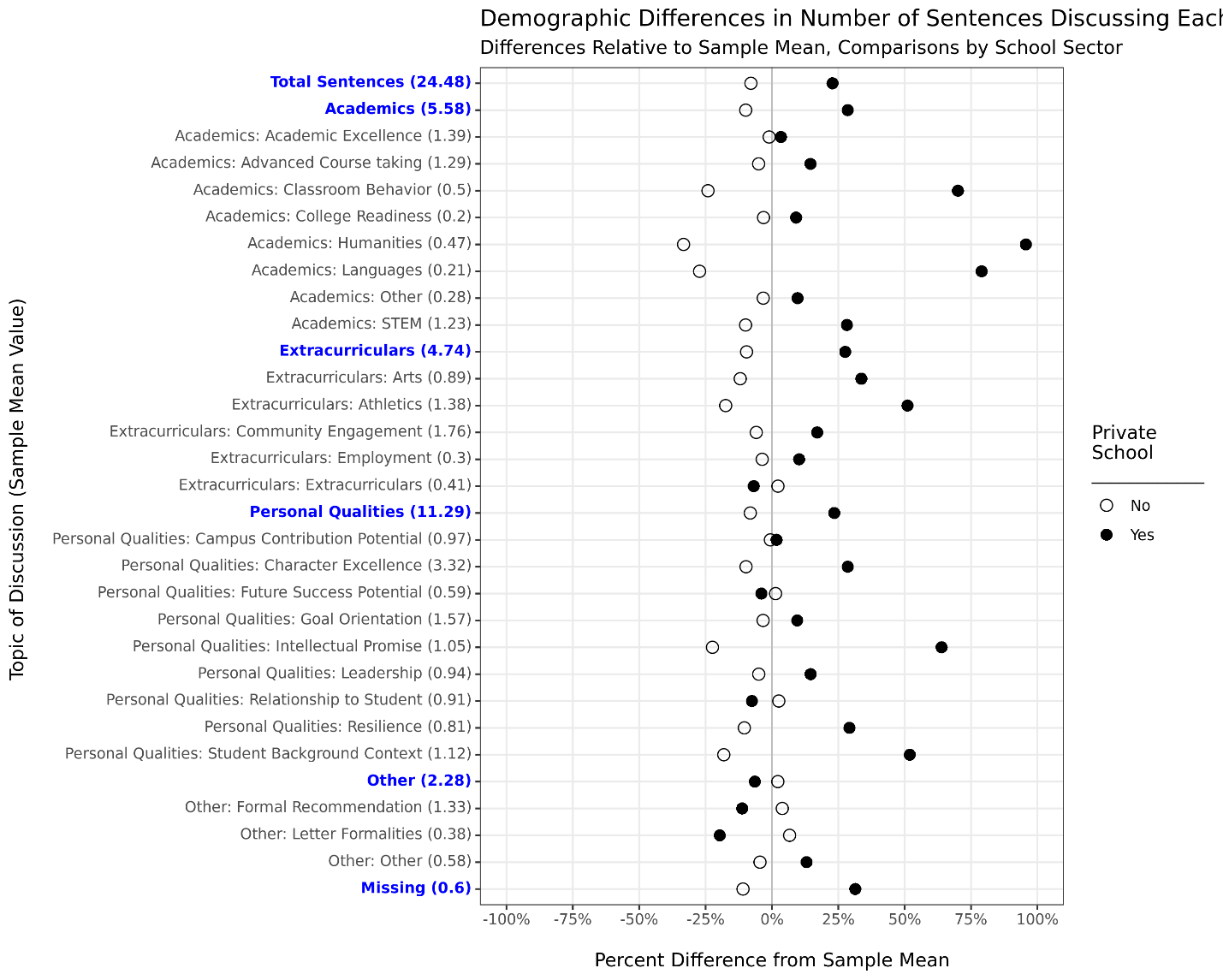

Supplement: Supplementary file 1 — Electronic supplementary material 1 (DOCX 1319 kb) [file 11162_2025_9847_MOESM1_ESM.docx]
